# PU.1 as a Master Regulator of Immune Cell Development and Function: Structural Basis, Gene Regulatory Networks, Disease Roles, and Therapeutic Targeting

**DOI:** 10.3390/biology15181657

**Published:** 2026-09-18

**Authors:** Mili Ramani, Ngoc Nguyen, Jamie Solis, Daniel G. Tenen, Bon Q. Trinh

**Affiliations:** 1 Department of Pathology, University of Virginia School of Medicine, Charlottesville, VA 22908, USA; qpf9my@virginia.edu (M.R.);; 2 Department of Life Sciences, Gachon University, Global Campus, Seongnam-si 13120, Republic of Korea; 3 Harvard Medical School Initiative for RNA Medicine, Harvard Medical School, Boston, MA 02115, USA; 4 Harvard Stem Cell Institute, Harvard Medical School, Boston, MA 02115, USA; 5 Center for RNA Science and Medicine, University of Virginia, Charlottesville, VA 22908, USA; 6 Molecular Genetics & Epigenetics Program, University of Virginia Comprehensive Cancer Center, Charlottesville, VA 22908, USA

**Keywords:** master regulator, immune cell, development and function, gene regulatory networks, disease roles, therapeutic targeting

## Abstract

PU.1 is a hematopoietic ETS-family transcription factor that serves as a master regulator of immune cell development, lineage commitment, chromatin organization, and innate immune function. PU.1 is essential for the generation and maintenance of monocytes, macrophages, dendritic cells, granulocytes, and B lymphocytes, while its repression is required for T-cell lineage commitment. As a lineage-determining transcription factor, PU.1 establishes cell identity by binding enhancer elements, recruiting chromatin remodelers, and cooperating with partner factors such as C/EBPα, RUNX1, IRF8, and GATA factors. Tight quantitative control of PU.1 is critical, as both reduced and excessive activity can disrupt hematopoiesis and immunity. Dysregulation of PU.1 has been implicated in acute myeloid leukemia (AML), myelodysplastic syndromes (MDS), lymphoid malignancies, chronic inflammatory disorders, fibrosis, and neurodegenerative disease. Recent structural and mechanistic advances have provided insights into the structure of PU.1, DNA recognition specificity, post-transcriptional regulation, and therapeutic vulnerabilities. In this review, we summarize current knowledge of PU.1 protein structure, regulatory mechanisms, binding partners, roles in disease, and emerging strategies to therapeutically target PU.1-centered networks.

## Simple Summary

The immune system depends on producing the right blood and immune cells in the right numbers. The regulatory protein, PU.1, helps determine what type of immune cell an immature blood cell will become and how that cell functions after maturation. The amount of PU.1 acts almost like a dial: different levels steer cells toward different fates, and maintaining PU.1 within an appropriate range is essential for normal function. When PU.1 levels fall too low or rise too high, the consequences can be serious, contributing to blood cancers such as leukemia, bone marrow failure, chronic inflammation, and changes in brain immune cells associated with neurodegenerative disease. This review brings together current knowledge of how PU.1 functions, how its levels are regulated, and why it has emerged as an attractive yet challenging therapeutic target, with strategies aimed at restoring its activity in some conditions while reducing or redirecting it in others.

## 1. Introduction

Precise control of gene expression is fundamental to immune cell development and functional specialization. Hematopoietic stem cells (HSCs) generate the full spectrum of mature blood cells through tightly coordinated lineage-specification programs directed by transcription factor networks, dynamic chromatin accessibility states, and extracellular signaling cues [1,2,3]. Among the central regulators of hematopoiesis, PU.1, encoded by *PU.1* (also called *SPI1*), occupies a uniquely important position because it governs both early cell-fate decisions and terminal effector functions across multiple immune lineages [1,4]. The human *PU.1* gene, located on chromosome 11p11.2, is primarily expressed in hematopoietic cells. Its expression is tightly controlled by proximal promoter and distal lineage-specific enhancers, which together integrate developmental signals to ensure context-dependent *PU.1* expression. Detailed regulation of PU.1 expression is beyond the scope of this review and has been covered extensively elsewhere [5].

PU.1 is a member of the ETS family of DNA-binding transcription factors and was originally identified through retroviral insertional mutagenesis studies [6,7]. Subsequent genetic and biochemical investigations established PU.1 as an essential regulator of both myeloid and lymphoid development. Mice lacking PU.1 fail to generate mature macrophages and B cells and exhibit profound defects in granulocyte differentiation [4]. Notably, PU.1 functions in a dosage-dependent manner: relatively high expression promotes macrophage development, intermediate levels support B-cell lineage commitment, and reduced expression predisposes to leukemogenesis and other hematologic malignancies [8,9,10,11].

Recent advances in epigenomics have expanded the view of PU.1 beyond that of a conventional transcription factor. PU.1 is now recognized as a pioneer factor capable of binding condensed chromatin and establishing enhancer landscapes required for lineage-specific gene expression [12,13]. Through cooperative interactions with partners such as C/EBP family members, IRFs, RUNX1, and related factors, PU.1 helps generate lineage-specific cistromes that define cellular identity and regulate responsiveness to inflammatory or environmental stimuli [14,15,16].

The biological significance of PU.1 extends beyond the hematopoietic system. Dysregulation of PU.1 has been implicated in a wide range of disease states, including acute myeloid leukemia (AML), myelodysplastic syndromes (MDS), lymphoid malignancies, chronic inflammation, autoimmunity, fibrotic remodeling, and neurodegenerative disorders associated with microglial dysfunction [17,18,19]. It is indispensable for microglial identity and maintenance in the central nervous system, underscoring its broader role in tissue-resident immune regulation [20,21]. At the same time, increasing structural insight into ETS-domain DNA recognition and protein–protein interaction interfaces has created new opportunities to therapeutically target PU.1 or its downstream transcriptional and epigenetic dependencies [22,23,24,25].

In this review, we synthesize current knowledge of the biological and mechanistic roles of PU.1, discuss its contributions to health and disease, and evaluate the emerging potential of targeting PU.1-driven regulatory circuits for clinical intervention. We first examine the molecular features of the PU.1 protein that underlie its regulatory capacity. In subsequent sections, we discuss how PU.1 regulates gene expression through promoter and enhancer binding, chromatin remodeling, and cooperative interactions with other transcription factors. We further explore indirect and non-canonical mechanisms of PU.1 action, its role in pathology, and emerging strategies to therapeutically target PU.1-driven transcriptional programs.

## 2. Methods

Our thorough literature search was conducted across multiple databases, including MEDLINE, PubMed Central, the NCBI Gene Expression Omnibus (GEO), and Embase. To identify studies investigating the diverse roles of PU.1 in immunology, hematology/oncology, and neurology, we used the following key search terms: “PU.1,” “SPI1,” “master regulator,” “pioneer transcription factor,” “ETS-family transcription factor,” “enhancer,” “promoter,” “cistrome,” “DNA recognition specificity,” “dosage-dependent regulation,” “RUNX1,” “C/EBP family,” “IRF8/IRF4,” “hematopoietic stem cells,” “lineage commitment,” “myeloid,” “lymphoid,” “monocytes/macrophages,” “dendritic cells,” “B lymphocytes,” “microglia,” “myelodysplastic syndromes,” “inflammation,” “neurodegenerative disease,” “Alzheimer’s disease,” “solid tumors,” “therapeutic targeting,” “BET,” “HDAC,” and “RNA therapeutics.” To capture the breadth of the relevant literature, the database search was supplemented by manual review of the reference lists of relevant studies (backward snowballing) and articles that subsequently cited them (forward snowballing).

Following this search, our inclusion criteria comprised peer-reviewed articles published through 2026, primarily reporting original research, supplemented by selected reviews and case reports. We focused on studies addressing the molecular, cellular, developmental, or disease-related aspects of *PU.1/SPI1* biology and therapeutic targeting across the contexts described above. From the pool of eligible publications, we prioritized studies that most directly aligned with the objectives of this review. Emphasis was placed on contemporary literature, while influential earlier studies were also incorporated. The resulting findings were organized into the subsequent sections.

## 3. Structural Basis and Functional Domains of PU.1

PU.1 is a DNA-binding nuclear protein of the ETS family that contains approximately 270 amino acids and is organized into three major functional regions: an N-terminal transactivation domain (TAD), a central proline/glutamic acid/serine/threonine (PEST)-rich regulatory region, and a C-terminal ETS DNA-binding domain [23,26]. This multidomain architecture enables PU.1 to integrate DNA recognition, cofactor recruitment, signal responsiveness, and chromatin remodeling.

### 3.1. The ETS DNA-Binding Domain

**Figure 1:**
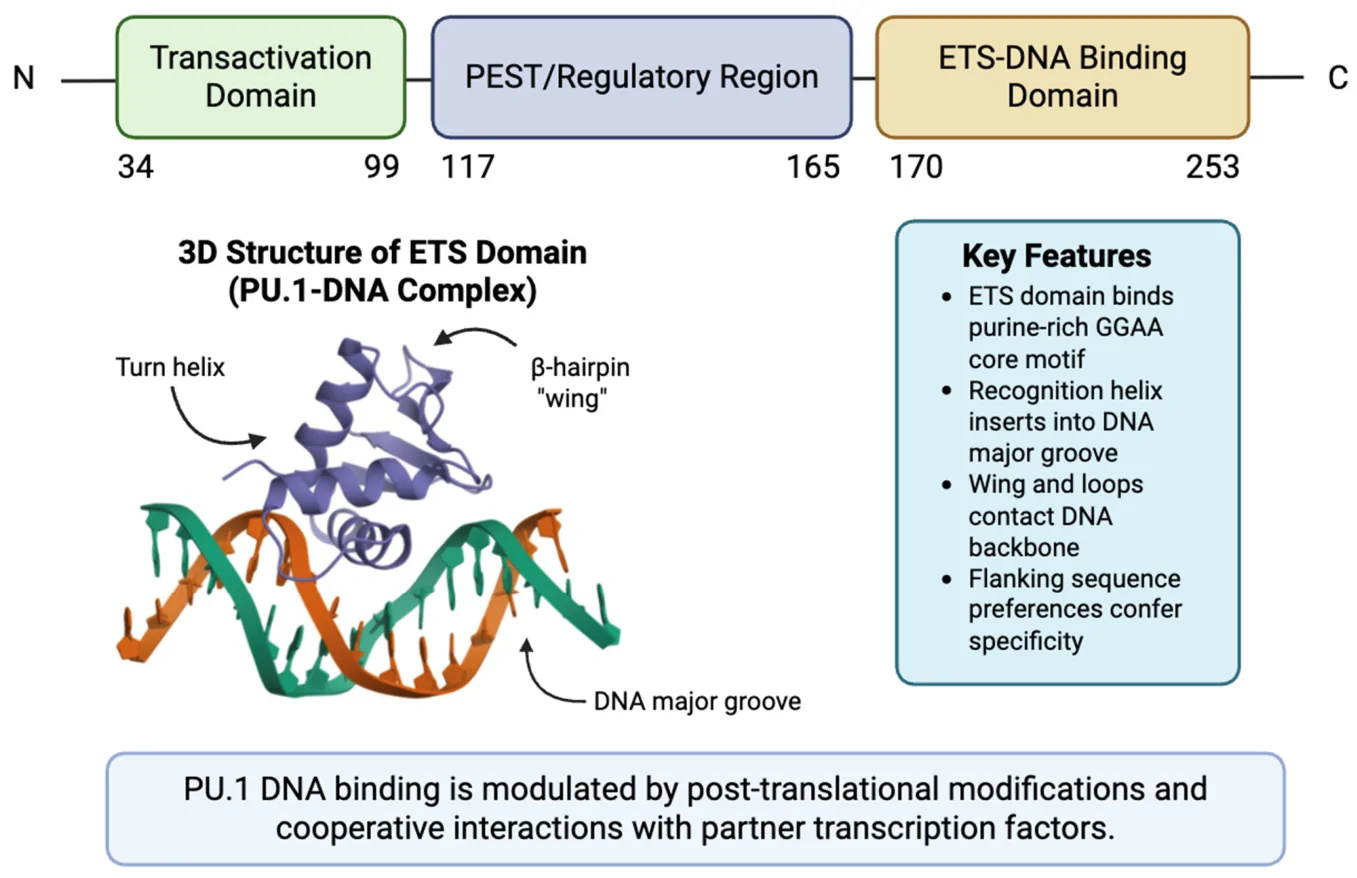
Structural domains of PU.1 and mechanisms of DNA binding. The primary protein structure comprises an N-terminal transactivation domain, a central PEST regulatory region, and a C-terminal ETS domain responsible for DNA binding. Of note, there are slight variations in domain boundaries among publications. The 3D complex highlights the interaction between the ETS domain (featuring a turn helix and β-hairpin wing) and the DNA major groove at purine-rich GGAA core motifs. This binding mechanism is further fine-tuned by flanking sequence specificity, post-translational modifications, and cooperative binding with transcriptional co-regulators.

The defining structural feature of PU.1 is the C-terminal ETS domain, which adopts a winged helix-turn-helix fold characteristic of ETS family transcription factors [23]. Within this domain, the recognition helix inserts into the major groove of DNA and mediates sequence-specific interactions with purine-rich motifs centered on the canonical 5′-GGAA-3′ core sequence, while adjacent loops and β-strands stabilize the protein–DNA interface (Figure 1). Structural studies have shown that PU.1 differs from several other ETS proteins by preferentially recognizing specific flanking nucleotides, particularly AT-rich neighboring bases, a property that likely contributes to lineage-selective enhancer occupancy in vivo [27].

DNA binding by PU.1 is not determined solely by the core motif sequence but is also influenced by motif affinity, local chromatin accessibility, cooperative interactions with nearby transcription factors, and intracellular PU.1 concentration [25,28]. In some contexts, PU.1 can bind DNA with alternative stoichiometries and induce local DNA bending or distortion, further expanding its regulatory versatility [29]. Importantly, mutations affecting ETS-domain residues can alter DNA-binding affinity or specificity and have been linked to malignant transformation, particularly in B-cell neoplasms and hematologic cancers [25,30].

Beyond direct DNA recognition, the ETS domain also mediates protein–protein interactions with other lineage-regulatory factors, including RUNX1, C/EBPα, C/EBPβ, GATA factors, c-Jun, and IRF family proteins, thereby enabling cooperative transcriptional regulation at composite cis-regulatory elements [31,32,33,34,35,36].

### 3.2. The Transactivation Domain

The N-terminal region of PU.1 functions as a transcriptional activation domain composed of multiple acidic and glutamine-rich subdomains that recruit components of the basal transcriptional machinery and chromatin-modifying complexes [23] (Figure 1). This region interacts with cofactors such as CBP/p300, TFIID, and Mediator [12,37,38,39,40].

The transactivation domain also contains regulatory serine residues that can be phosphorylated in response to signaling pathways, modulating PU.1 activity in a context-dependent manner [40,41]. Such post-translational modifications have been implicated in macrophage proliferation, cytokine responsiveness, and induction of genes such as the macrophage colony-stimulating factor 1 receptor (*CSF1R*) [41,42,43].

### 3.3. The PEST Regulatory Region

Located between the transactivation and ETS domains, the central PEST-rich region is enriched in proline, glutamic acid, serine, and threonine residues and serves as an important regulatory hub [23] (Figure 1). Although PEST motifs are commonly associated with rapid protein turnover [44], in PU.1 this region also functions as a platform for protein–protein interactions and signal integration. It is subject to phosphorylation, ubiquitination, and proteasomal degradation pathways, allowing extracellular cues to dynamically regulate PU.1 abundance and activity [45,46].

Conversely, PU.1 stability is actively maintained by deubiquitination: the deubiquitinase USP7 directly binds and deubiquitinates PU.1, shielding it from proteasomal degradation. In murine HSCs, conditional deletion of *Usp7* destabilizes PU.1 and suppresses PU.1 target genes required for quiescence and lineage specification, resulting in defective self-renewal, multilineage cytopenias, and hematopoietic failure; ectopic *PU.1* expression partially rescues this phenotype, defining a USP7-PU.1 axis that is critical for stem-cell integrity [47].

The PEST region also contributes to interactions with interferon regulatory factors such as IRF4 and IRF8 and can influence recruitment of cofactors to composite DNA elements [48]. Interestingly, deletion or alteration of this region can enhance expression of certain monocyte-specific genes, suggesting that the PEST domain may also exert context-dependent inhibitory control over PU.1 transcriptional activity [49].

### 3.4. Functional Integration of PU.1 Domains

The coordinated action of these three domains allows PU.1 to function as more than a simple sequence-specific transcription factor. The ETS domain confers selective DNA recognition, the transactivation domain recruits transcriptional and chromatin-remodeling machinery, and the PEST region integrates signaling-dependent regulation and cofactor interactions [12]. Together, these features enable PU.1 to establish and maintain lineage-specific chromatin accessibility landscapes, regulate immune cell identity, and coordinate responses to developmental or inflammatory cues [28,39]. Disruption of any of these integrated molecular functions therefore can profoundly alter cellular differentiation and contribute to leukemogenesis, immune dysregulation, and neuroinflammatory disease, which will be elaborated in respective sections.

## 4. Post-Transcriptional Regulation of PU.1

The *PU.1* mRNA transcript contains regulatory untranslated regions that influence mRNA stability, subcellular localization, and translational efficiency [14]. These features provide an additional layer of control over PU.1 protein abundance. Post-transcriptional regulation is mediated by several microRNAs, including miR-155 and miR-150, which directly target the *PU.1* 3′UTR to suppress its expression in lineage- and context-specific manners [50,51]. Through modulation of PU.1 dosage, these microRNAs shape hematopoietic differentiation, inflammatory responses, and leukemogenic programs. Conversely, PU.1 itself transcriptionally activates miR-233 and the miR-23a cluster [52,53,54]. These microRNAs act as downstream effectors to coordinate myeloid differentiation with cell cycle arrest and to antagonize lymphoid cell fate [53,54]. In addition, RNA-binding proteins are likely to influence *PU.1* transcript splicing, stability, and translation [14,55,56], although these mechanisms remain comparatively understudied.

Beyond transcript-level regulation, PU.1 activity is extensively modulated after translation through multiple post-translational modifications that rapidly tune its function in response to developmental and environmental cues. Phosphorylation is one of the best-characterized mechanisms and can alter DNA-binding affinity, cofactor recruitment, and transcriptional output. For example, phosphorylation of Ser148 promotes interaction with the cofactor IRF4 (NF-EM5), supporting full transcriptional activation [40], whereas AKT-mediated phosphorylation within the transactivation domain, including at Ser41, enhances PU.1 activity [57]. These findings suggest that signaling pathways can directly couple extracellular stimuli to PU.1-dependent gene regulation.

Additional modifications further diversify PU.1 control. Acetylation has been reported to enhance transcriptional activity, potentially by promoting cofactor recruitment or increasing chromatin accessibility at target loci [58,59]. Ubiquitination regulates protein stability and turnover, allowing rapid adjustment of intracellular PU.1 levels during lineage commitment or immune activation [45].

**Figure 2:**
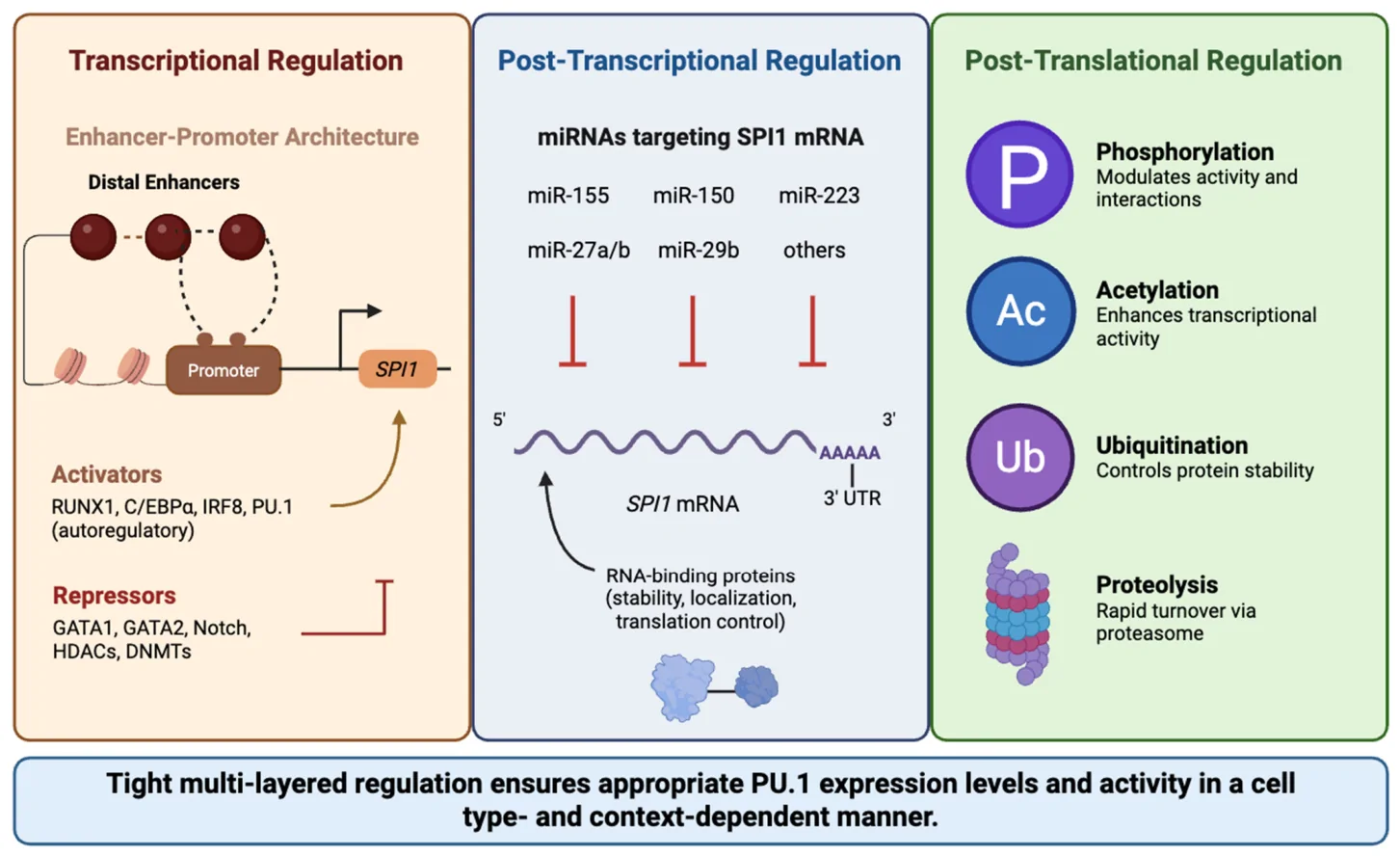
Regulation of PU.1 expression. *PU.1* expression is modulated at the gene level by distal enhancer-promoter looping, positive transcription factors (e.g., RUNX1, C/EBPα and autoregulatory PU.1), and negative epigenetic or transcriptional repressors (e.g., GATA factors, DNMTs). Post-transcriptionally, *PU.1* mRNA is targeted by key microRNAs (e.g., miR-155, miR-150) and RNA-binding proteins to control translation and stability. Post-translationally, PU.1 protein activity and turnover are dynamically controlled by phosphorylation, acetylation, and ubiquitin-mediated proteasomal degradation.

Collectively, these post-transcriptional and post-translational regulatory mechanisms provide a dynamic framework for fine-tuning PU.1 function beyond simple control of gene expression (Figure 2). By integrating microRNA networks, RNA metabolism, and signaling-dependent protein modifications, myeloid cells can rapidly adapt PU.1 activity during hematopoietic differentiation, immune activation, and stress responses.

## 5. PU.1 as a Master Regulator of Gene Expression, Enhancers, and Chromatin Architecture

PU.1 controls hematopoietic lineage specification and immune-cell function through diverse mechanisms that extend far beyond conventional promoter activation. In addition to regulating protein-coding genes, PU.1 modulates noncoding RNAs and autoregulatory circuits [60,61,62]. It also regulates enhancer activity, chromatin accessibility, and higher-order three-dimensional genome organization [12,39]. These integrated functions establish PU.1 as a master regulator of myeloid and lymphoid identity.

### 5.1. Promoter Binding and Direct Transcriptional Activation

PU.1 binds promoter regions of numerous genes essential for hematopoietic development and immune signaling. Classical PU.1 target genes include cytokine receptor loci such as *CSF1R* (M-CSF receptor) [43,63], *CSF2RA* (GM-CSF receptor α) [63,64], and *CSF3R* (G-CSF receptor) [63,65]. PU.1 recruits transcriptional machinery to these loci to stimulate expression programs required for myeloid differentiation. Through regulation of these receptors, PU.1 links intrinsic lineage-specifying transcriptional programs to extrinsic environmental signals, ensuring that progenitors acquire appropriate responsiveness to growth factors, inflammatory cytokines, and survival cues during development. PU.1 also activates additional lineage-restricted genes involved in antigen presentation, phagocytosis, pattern recognition, and innate immune signaling [62,63], thereby coordinating both developmental identity and mature effector function.

### 5.2. Autoregulation and Regulatory Feedback Loops

*PU.1* expression is itself tightly controlled through autoregulatory mechanisms. The *PU.1* locus resides within a dynamic chromatin domain in which promoter and distal enhancer elements physically interact during myeloid differentiation. PU.1 can bind its own regulatory elements to reinforce its RNA transcription, thereby stabilizing lineage commitment once a threshold level of expression has been achieved [61,66]. This positive autoregulation is supported by chromatin adaptors such as LDB1 and contributes to the formation of a transcriptionally active three-dimensional architecture at the *PU.1* locus [67]. Such autoregulatory feedback likely explains how modest early increases in *PU.1* expression can be converted into stable lineage decisions, particularly during monocyte/macrophage differentiation [68]. Conversely, disruption of these loops may contribute to reduced *PU.1* expression in leukemia and other hematologic disorders [19].

### 5.3. Regulation of Noncoding RNAs

**Figure 3:**
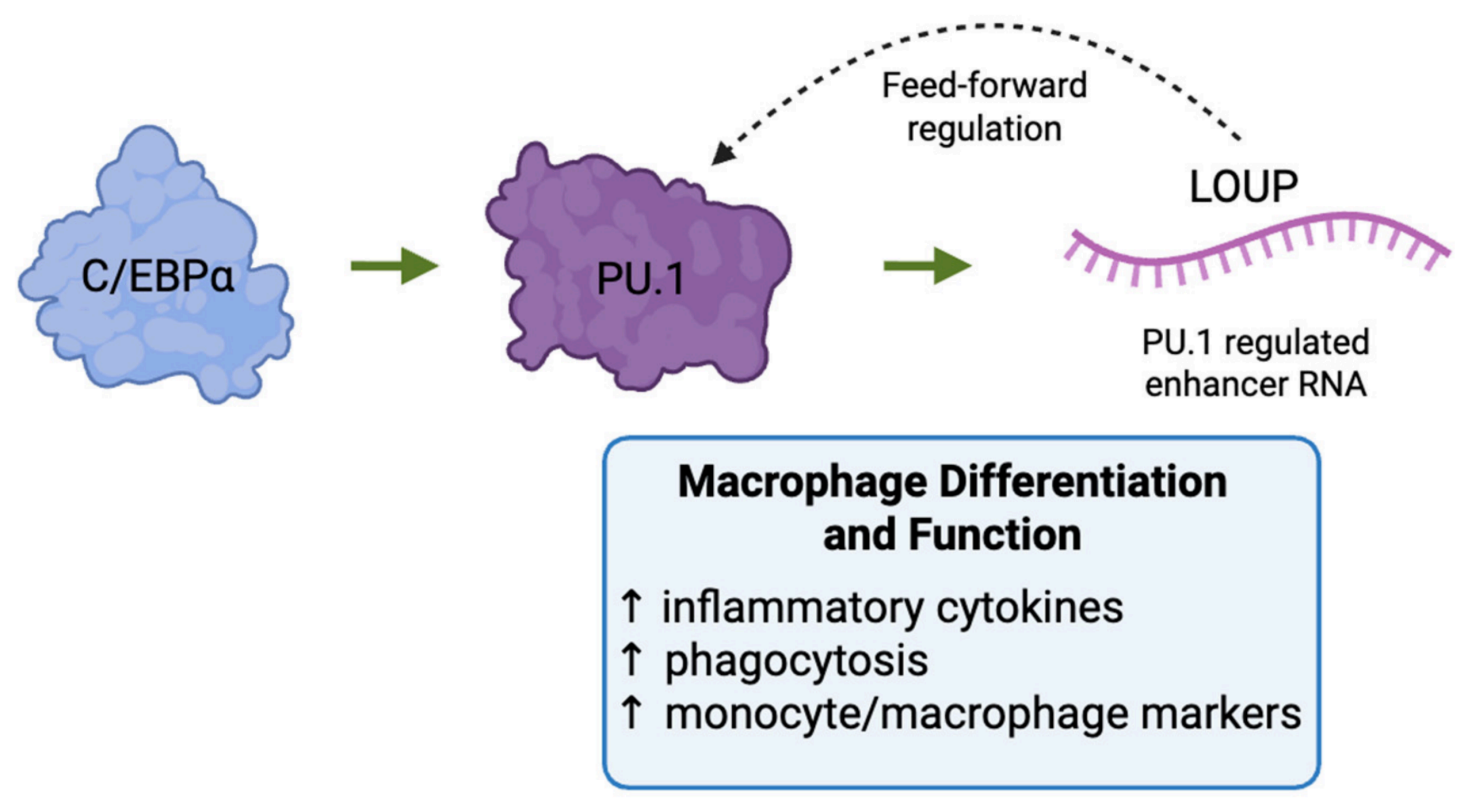
The PU.1-LOUP Feed-Forward Regulatory Circuit. Upstream transcription factor C/EBPα stimulates PU.1, leading to the production of the PU.1-regulated enhancer RNA, *LOUP*. *LOUP* participates in a positive feed-forward regulatory mechanism targeting PU.1 to solidify myeloid identity. The transcriptional loop ultimately coordinates macrophage differentiation and immune functions, including the upregulation of monocyte/macrophage surface markers, increased phagocytosis, and elevated production of inflammatory cytokines.

Emerging evidence indicates that PU.1 also controls gene expression through noncoding RNA networks. PU.1 regulates long noncoding RNAs (lncRNAs), including enhancer RNAs (eRNAs) [39,62,69,70,71], and microRNAs [69] that reinforce cell identity or inflammatory responses. A notable example is *LOUP*, a myeloid-restricted eRNA induced within a PU.1-regulated enhancer cluster. *LOUP* participates in a feed-forward circuit with C/EBPα and PU.1, in which C/EBPα promotes *PU.1* expression, PU.1 induces *LOUP*, and *LOUP* reinforces myeloid differentiation and macrophage function (Figure 3) [62,70,71]. Depletion of *LOUP* impairs monocyte/macrophage marker expression, inflammatory cytokine production, and phagocytic activity [62]. These findings suggest that PU.1 regulates not only coding genes but also RNA-based regulatory systems that amplify and stabilize lineage-specific transcriptional states.

### 5.4. PU.1 as a Pioneer Factor and Enhancer Selector

Recent epigenomic and single-cell studies have redefined PU.1 as a pioneer transcription factor that establishes lineage-specific enhancer landscapes. PU.1 can engage partially inaccessible chromatin, recognize nucleosomal DNA, recruit remodeling complexes, and generate permissive enhancer states during macrophage, dendritic-cell, eosinophil, and B-cell development. Unlike some classical pioneer factors, efficient pioneering by PU.1 often requires not only its ETS DNA-binding domain but also its acidic transactivation domain and intrinsically disordered regions [13,28]. These domains facilitate recruitment of ATP-dependent chromatin remodelers and stabilization of chromatin opening [12]. Once engaged, PU.1 marks enhancers with active histone modifications such as H3K4me1 and H3K27ac [12], priming them for rapid activation. Primed enhancers can be activated by signal-dependent factors including NF-κB [12,39], AP-1 [15,72], and IRFs [73,74]. Genome-wide studies show that distinct myeloid cell types possess unique repertoires of PU.1-bound enhancers [12,28], suggesting that PU.1 collaborates with local cofactors to generate cell type-specific enhancer selection. Thus, PU.1 serves as both a lineage determinant and a platform for context-specific inflammatory signaling.

### 5.5. Chromatin Remodeling and Polycomb Antagonism

PU.1 plays a central role in maintaining accessible chromatin landscapes. Structural studies have shown that PU.1 can bind enhancer motifs near nucleosome entry/exit sites, partially unwrap nucleosomal DNA, and destabilize compact chromatin structures [13,75]. Cooperative binding with factors such as C/EBPα further enhances nucleosome opening and displacement of linker histone H1, promoting broader accessibility [75]. PU.1 also recruits SWI/SNF chromatin-remodeling complexes through direct interactions with its transactivation domain [76]. These remodelers reposition nucleosomes and facilitate access of secondary transcription factors [12,28]. In leukemia cells, PU.1-dependent recruitment of SWI/SNF complexes helps sustain oncogenic regulatory circuits, whereas SWI/SNF inhibition can redistribute PU.1 binding and promote differentiation [76]. In parallel, PU.1 antagonizes Polycomb repressive complex 2 (PRC2), preventing deposition of repressive H3K27me3 marks at myeloid enhancers. In the absence of PU.1, these loci can become heterochromatic and inaccessible [77]. Therefore, PU.1 not only activates enhancers but also protects lineage-specific regulatory regions from epigenetic silencing.

### 5.6. Enhancer–Promoter Looping and Three-Dimensional Genome Organization

PU.1 also functions as a higher-order regulator of chromatin architecture. At inflammatory loci such as *IL1B*, PU.1 binding to distal enhancers induces nucleosome depletion, enhancer-promoter looping, and H3K27ac, creating a poised chromatin state for rapid transcriptional activation upon stimulation [39,62]. These events are independently required for subsequent NF-κB recruitment and eRNA transcription during innate immune activation [39,62]. More broadly, PU.1 contributes to formation and maintenance of lineage-specific chromosomal interactions within sub-topologically associating domains (subTADs) [67]. Cooperation with architectural complexes further shapes PU.1-dependent enhancer function. In conventional dendritic cells, the cohesin complex, through its SMC3 and STAG2 subunits, promotes PU.1 binding at super-enhancers and supports chromatin looping. These cohesin-dependent interactions help establish the cDC-specific gene expression program, linking PU.1-mediated enhancer selection to higher-order genome folding [78]. Through such mechanisms, PU.1 coordinates spatial proximity between enhancers and promoters, enabling precise temporal control of transcription during differentiation and immune responses [16]. Genetic variants that disrupt PU.1-dependent looping at neutrophil enhancers have been associated with autoimmune disease susceptibility [79], highlighting the functional importance of these three-dimensional interactions.

### 5.7. Functional Significance

Collectively, PU.1 regulates gene expression through multiple interconnected layers: direct promoter activation, autoregulation, control of noncoding RNAs, enhancer priming, chromatin remodeling, Polycomb exclusion, and enhancer-promoter looping. These integrated mechanisms allow PU.1 to synchronize intrinsic developmental programs with extrinsic cytokine and inflammatory signals [80]. As a result, PU.1 is indispensable for myeloid differentiation, rapid innate immune responses, and long-term maintenance of lineage identity [81,82]. Disruption of any of these regulatory functions can contribute to leukemia, immune dysfunction, chronic inflammation, and neurodegenerative disease.

## 6. PU.1 in Cooperative Transcription Factor Networks and Gene Regulatory Circuits

PU.1 rarely functions in isolation. Instead, its biological activity emerges through combinatorial interactions with other transcription factors, cofactors, and chromatin regulators that together establish lineage-specific gene expression programs [14]. Through these partnerships, PU.1 acts as both a DNA-binding factor and a molecular scaffold that coordinates enhancer selection, chromatin remodeling, and transcriptional output. Such cooperative networks are essential for hematopoietic lineage commitment, cellular plasticity, and immune responsiveness (Table 1).

At the structural level, these interactions are enabled by multiple PU.1 domains. The conserved ETS domain binds GGAA-containing DNA motifs and mediates contacts with transcription factors including GATA-1, GATA-2, RUNX1, C/EBPα, C/EBPβ, c-Jun, and IRF family proteins [31,32,33,34,35,36]. The N-terminal acidic and glutamine-rich regions support transcriptional activation, whereas the central PEST domain contributes to selective cofactor recruitment, particularly with IRF4 and IRF8 [48,83]. Together, these domains allow PU.1 to integrate multiple signaling and lineage-determining inputs into coherent transcriptional programs.

### 6.1. The PU.1–C/EBP Axis

One of the best-characterized PU.1 partnerships is with C/EBPs, particularly C/EBPα and C/EBPβ. These factors cooperate extensively to establish myeloid enhancer landscapes and activate macrophage differentiation programs [62,75]. Genome-wide studies demonstrate strong co-occupancy of PU.1 and C/EBP factors at monocyte/macrophage enhancers, where they jointly recruit chromatin remodelers and transcriptional coactivators. Recent structural studies have shown that PU.1 and C/EBPα can bind cooperatively to nucleosomal DNA, jointly unwrapping approximately 45 base pairs of nucleosomal DNA and displacing linker histone H1. This coordinated action opens compact chromatin and permits assembly of additional regulatory complexes [75]. Such cooperative pioneering is critical for establishing monocyte-specific enhancers and enabling downstream responses to inflammatory signaling pathways such as NF-κB activation. The PU.1–C/EBP relationship also operates through feed-forward regulatory loops. C/EBPα activates a distal enhancer upstream of the *PU.1* locus, thereby promoting *PU.1* expression and monocyte lineage commitment. In turn, PU.1 activates myeloid targets including the enhancer RNA *LOUP*, which reinforces the C/EBPα–PU.1 circuit and supports macrophage differentiation and innate immune function [62]. However, the interaction is context-dependent. In some settings, C/EBPα can antagonize PU.1 by competing for cofactors such as c-Jun, thereby favoring granulocytic rather than monocytic differentiation [82,84]. Thus, PU.1 and C/EBP factors can be cooperative or competitive depending on developmental stage and signaling environment.

### 6.2. The PU.1–IRF Axis

PU.1 also collaborates extensively with interferon regulatory factors (IRFs), especially IRF4 and IRF8. Composite ETS–IRF DNA motifs enable cooperative binding of PU.1 and IRFs at regulatory regions [36,73,85,86,87]. This cooperation controls gene programs involved in dendritic-cell development, B-cell maturation, antigen presentation, and immune activation. PU.1 often functions as a pioneer or anchoring factor that helps recruit IRF proteins to specific genomic loci. Moreover, scaffolding proteins may contribute to this regulation. TopBP1, a BRCT-domain protein best known for its roles in the ATR-dependent DNA-damage checkpoint and V(D)J recombination, directly binds the PU.1–IRF8 heterodimer and promotes transcription of genes required for conventional dendritic-cell specification [88]. This finding reveals a non-canonical, DNA-repair-independent role for TopBP1 as a co-regulator of these lineage-defining factors. Chromatin immunoprecipitation studies have shown co-occupancy of PU.1 and IRF8 at enhancers and promoters of genes such as *Pax5*, immunoglobulin light-chain loci, and *Mef2c*, suggesting that PU.1 directs IRF4/IRF8 to lineage-relevant targets [36,89,90]. These interactions are particularly important in dendritic-cell subset specification and B-cell gene regulation. Because IRF proteins are major mediators of interferon signaling and pathogen responses, the PU.1–IRF axis also links developmental identity with inducible immune transcriptional programs.

### 6.3. The PU.1–RUNX1 Axis

RUNX1 is both an upstream regulator and functional partner of PU.1. RUNX1 binds regulatory elements within the *PU.1* locus to support PU.1 expression during hematopoietic differentiation [70,91,92]. Beyond this transcriptional control, RUNX1 cooperates with PU.1 at target genes by facilitating exchange of corepressors for coactivators, thereby enabling activation of genes such as *CSF1R* and *CSF2RA* [33,93]. Loss of RUNX1 function or expression of leukemia-associated truncated RUNX1 variants can impair these cooperative interactions, increase repressive complexes at PU.1-bound loci, and block differentiation [33,93,94]. These observations suggest that defective RUNX1–PU.1 cooperation contributes to leukemogenesis and may represent a targetable vulnerability in myeloid malignancies.

### 6.4. Cooperation with AP-1 Family Factors

PU.1 also interacts with c-Jun and related AP-1 family proteins. The c-Jun interaction surface lies within the ETS domain of PU.1. Functional studies using PU.1 mutants that retain DNA-binding ability but cannot bind c-Jun demonstrated that this physical interaction is required for efficient monocyte/macrophage differentiation [31,95]. Restoration of c-Jun tethering rescues differentiation, indicating that protein–protein interaction rather than DNA binding alone is critical. These findings underscore that PU.1 often requires partner factors to fully activate lineage programs. In macrophages, PU.1–AP-1 cooperation may also contribute to inflammatory gene induction downstream of Toll-like receptor signaling and NF-κB activation [15,72].

### 6.5. Antagonism with GATA Factors

The mutual antagonism between PU.1 and GATA factors, particularly GATA-1, represents a classic model of binary lineage choice between erythroid/megakaryocytic and myeloid fates. PU.1 and GATA-1 can physically interact and repress one another’s transcriptional activity [96,97]. GATA-1 inhibits PU.1-mediated activation partly by preventing recruitment of c-Jun and associated coactivators [96,98]. Conversely, PU.1 suppresses GATA-1 function by occupying GATA-bound loci and promoting repressive chromatin states [97]. GATA-2 also participates in this circuitry during early progenitor stages [99]. Reduction in GATA factor activity can increase PU.1 expression and reprogram cells toward macrophage differentiation [97], highlighting the dose-sensitive balance between these competing lineage determinants. This reciprocal repression model explains how hematopoietic progenitors commit decisively to alternative fates through reinforcement of one transcriptional network and silencing of the other.

### 6.6. PU.1 in Transdifferentiation and Cellular Plasticity

The power of PU.1-centered networks is illustrated by transdifferentiation studies. Forced PU.1 expression in megakaryocyte/erythroid progenitors can redirect cells toward myeloid identities [100], whereas GATA-1 expression in myeloid cells can drive erythroid or megakaryocytic conversion [98]. Likewise, expression of C/EBPα or C/EBPβ in pre-B [101] or pre-T [102] cells can generate macrophage-like cells in a process dependent on endogenous PU.1. These findings demonstrate that PU.1 is not merely a marker of lineage identity but a causal determinant capable of reshaping chromatin landscapes and cellular fate when placed within permissive transcription factor networks.

### 6.7. Functional Significance of Cooperative Networks

PU.1 functions as a central node in interconnected transcription factor circuits rather than as a solitary regulator. Cooperative interactions with C/EBP factors, IRFs, RUNX1, and AP-1 proteins promote myeloid and immune gene programs, whereas antagonism with GATA factors restricts erythroid and megakaryocytic pathways [97,99]. Through these dynamic partnerships, PU.1 integrates developmental signals, cytokine responses, and chromatin state to govern lineage commitment, immune activation, and cellular plasticity [80,103]. Disruption of these networks contributes directly to leukemia, immune deficiency, chronic inflammation, and aberrant lineage reprogramming [19,104].

## 7. PU.1 Functions Across Hematopoietic Lineages and Immune Cell Development

Expressed dynamically across the hematopoietic hierarchy, PU.1 influences multiple branches of blood development through dosage-sensitive and stage-specific mechanisms [7,81,105]. Rather than functioning as a simple on/off determinant, PU.1 acts as a quantitative lineage “rheostat,” whereby graded expression levels bias progenitors toward distinct developmental outcomes. This model highlights how relatively small changes in transcription factor abundance can generate substantial cellular diversity within the immune system. Low levels of PU.1 are present in hematopoietic stem cells (HSCs) and early multipotent progenitors, where they contribute to lineage priming while preserving developmental flexibility. Progressive increases in PU.1 expression reinforce commitment to myeloid and B-cell lineages [10]. In contrast, sustained repression of PU.1 is required for erythroid, megakaryocytic, and committed T-cell differentiation [99,106,107]. Thus, PU.1 functions both as a promoter of selected lineages and as an active suppressor of alternative fates.

### 7.1. Hematopoietic Stem Cells and Progenitors

PU.1 plays a foundational role in maintaining the HSC pool and guiding differentiation into downstream progenitor populations. In stem and early progenitor compartments, low-level PU.1 expression supports lineage priming, particularly toward myeloid programs, without fully extinguishing alternative potentials [81]. This poised state allows HSCs to respond flexibly to physiological demands such as infection, inflammation, or hematopoietic stress. As progenitors progress into common myeloid progenitors (CMPs), granulocyte–monocyte progenitors (GMPs), and common lymphoid progenitors (CLPs), PU.1 levels become increasingly informative for lineage direction (Figure 3). Perturbations in PU.1 dosage at these stages can skew hematopoietic output, leading to excessive myelopoiesis, impaired lymphopoiesis, or developmental arrest [81,108,109]. PU.1 activity in this compartment is also responsive to systemic metabolic state. Epigenetic profiling of HSCs from calorically restricted mice showed that caloric restriction remodels PU.1 genomic occupancy across thousands of sites, increasing PU.1 binding at genes involved in cell-cycle regulation, differentiation, and self-renewal. Through this mechanism, PU.1 redirects HSCs toward myeloid and thrombo-erythroid differentiation rather than self-renewal, while the endothelial receptor KDR (VEGFR2) emerged as a parallel regulator whose knockdown recapitulated the transcriptomic state induced by lifelong caloric restriction [110]. Notably, the functional importance of PU.1 dosage in this compartment is further reinforced by the post-translational mechanisms discussed earlier (Section 3.3). USP7-mediated deubiquitination stabilizes PU.1 and sustains expression of target genes required for HSC quiescence, whereas loss of this stabilizing mechanism destabilizes PU.1, resulting in defective quiescence, impaired self-renewal, and multilineage hematopoietic failure [47].

### 7.2. Monocytes and Macrophages

Among hematopoietic lineages, PU.1 is most strongly associated with monocyte and macrophage development. High *PU.1* expression promotes macrophage differentiation in fetal liver progenitors, bone marrow precursors, and multipotent cell models [10,100,111]. Mechanistically, PU.1 activates genes required for macrophage identity and innate immune function, including *CSF1R* (M-CSF receptor), Fc receptors, Toll-like receptor signaling components, phagocytic receptors, lysosomal enzymes, and inflammatory cytokines [43,112]. PU.1 remains essential after lineage commitment. Sustained expression in mature macrophages supports survival, proliferative potential, phagocytosis, and rapid inflammatory responses. Loss of PU.1 in differentiated macrophages compromises lineage identity and innate immune competence [42,82,113,114].

### 7.3. Dendritic Cells

PU.1 is indispensable for dendritic-cell (DC) development, including both conventional DCs (cDCs) and plasmacytoid DCs (pDCs). In these lineages, PU.1 cooperates with IRF4, IRF8, STAT proteins, and other regulators to establish subset-specific enhancer landscapes and transcriptional circuits [73,74,115]. The establishment of these enhancer landscapes also depends on chromatin architecture. Cohesin promotes PU.1 occupancy at super-enhancers to reinforce cDC identity, such that loss of the cohesin subunits SMC3 or STAG2 in DC-restricted progenitors reduces the cDC compartment, expands pDCs, de-represses the pDC transcriptional program within cDCs, and impairs antigen presentation and co-stimulatory molecule expression [78]. Furthermore, DC-specific deletion of TopBP1 in mice causes cDC deficiency with pre-DC accumulation, impairs Flt3L-driven terminal differentiation of cDC1s, and abrogates the efficacy of Flt3L-based antitumor immunotherapy, linking PU.1–IRF8 transcriptional programming to dendritic-cell-mediated tumor immunity [88]. PU.1 occupancy correlates with epigenetic remodeling during DC differentiation and helps drive expression of genes involved in antigen processing and cytokine production [116,117,118]. Given the specialized immune surveillance role of DCs, PU.1 is therefore critical not only for lineage specification but also for adaptive immune initiation.

### 7.4. Granulocytes and Neutrophils

PU.1 also contributes to granulocyte development, particularly in dosage-sensitive contexts. Intermediate *PU.1* expression can favor granulocytic differentiation, whereas excessively high levels tend to shift progenitors toward macrophage fates [108,111]. During neutrophil maturation, PU.1 regulates genes involved in adhesion, microbial killing, receptor signaling, and granule function [79,119,120]. Its role in granulopoiesis is closely integrated with C/EBP family transcription factors, especially C/EBPα and C/EBPε, which help determine neutrophil-specific maturation programs [121,122]. Thus, PU.1 dosage and partner-factor availability together influence the balance between granulocytic and monocytic development.

### 7.5. B Cells

*PU.1* is expressed in B cells and their precursors, including pro-B cells, where it supports early B-lineage specification and identity [10,123]. Intermediate *PU.1* expression appears optimal for B-cell development, whereas insufficient PU.1 impairs early B lymphopoiesis and excessive PU.1 can redirect progenitors toward myeloid fates [10,123]. PU.1 therefore functions as a finely tuned regulator within the B-cell developmental network.

### 7.6. T Cells

In contrast to myeloid and B-cell lineages, PU.1 must be repressed for efficient committed T-cell maturation [124]. Although transient PU.1 expression may occur in early lymphoid progenitors, continued expression interferes with T-lineage progression and can preserve alternative developmental potentials. Silencing of PU.1 is therefore an important checkpoint during thymocyte differentiation, enabling commitment to the T-cell program [107,125]. This requirement illustrates that PU.1 is not universally beneficial across hematopoiesis; rather, its developmental effects depend on precise lineage context and timing.

### 7.7. Additional Hematopoietic and Tissue-Resident Cell Lineages

PU.1 also contributes to mast-cell development and additional specialized hematopoietic subsets where it influences maturation and functional identity. Beyond the bone marrow, PU.1 has a particularly important role in microglia, the resident macrophages of the central nervous system. PU.1 is highly expressed in microglia and is required for their viability, homeostasis, and phagocytic function [126,127]. Experimental reduction in PU.1 impairs uptake of amyloid-β peptides and alters expression of genes linked to antigen presentation and neurodegenerative disease risk [22,128,129]. PU.1 directly regulates gene loci such as *TREM2* [126], highlighting its importance in microglial biology and neuroimmune regulation.

### 7.8. PU.1 as a Determinant of Hematopoietic Balance

PU.1 acts across the hematopoietic system to sustain stem-cell competence, guide lineage commitment, maintain differentiated cell identity, and coordinate immune function. High PU.1 levels generally favor macrophage programs, intermediate levels support granulocytic or B-cell pathways depending on context, and low or absent PU.1 permits erythroid, megakaryocytic, or T-cell progression [7,10,11,111]. Temporal and quantitative regulation of PU.1 is therefore essential for balanced hematopoietic output and immune homeostasis. Consistent with this, metabolic inputs such as caloric restriction can reshape PU.1 occupancy at target genes, biasing HSCs toward myeloid and thrombo-erythroid output at the expense of self-renewal and lymphopoiesis [110]. Disruption of this equilibrium can lead to immune deficiency [104], chronic inflammation [79], lineage skewing and hematologic malignancy [9,19].

**Figure 4:**
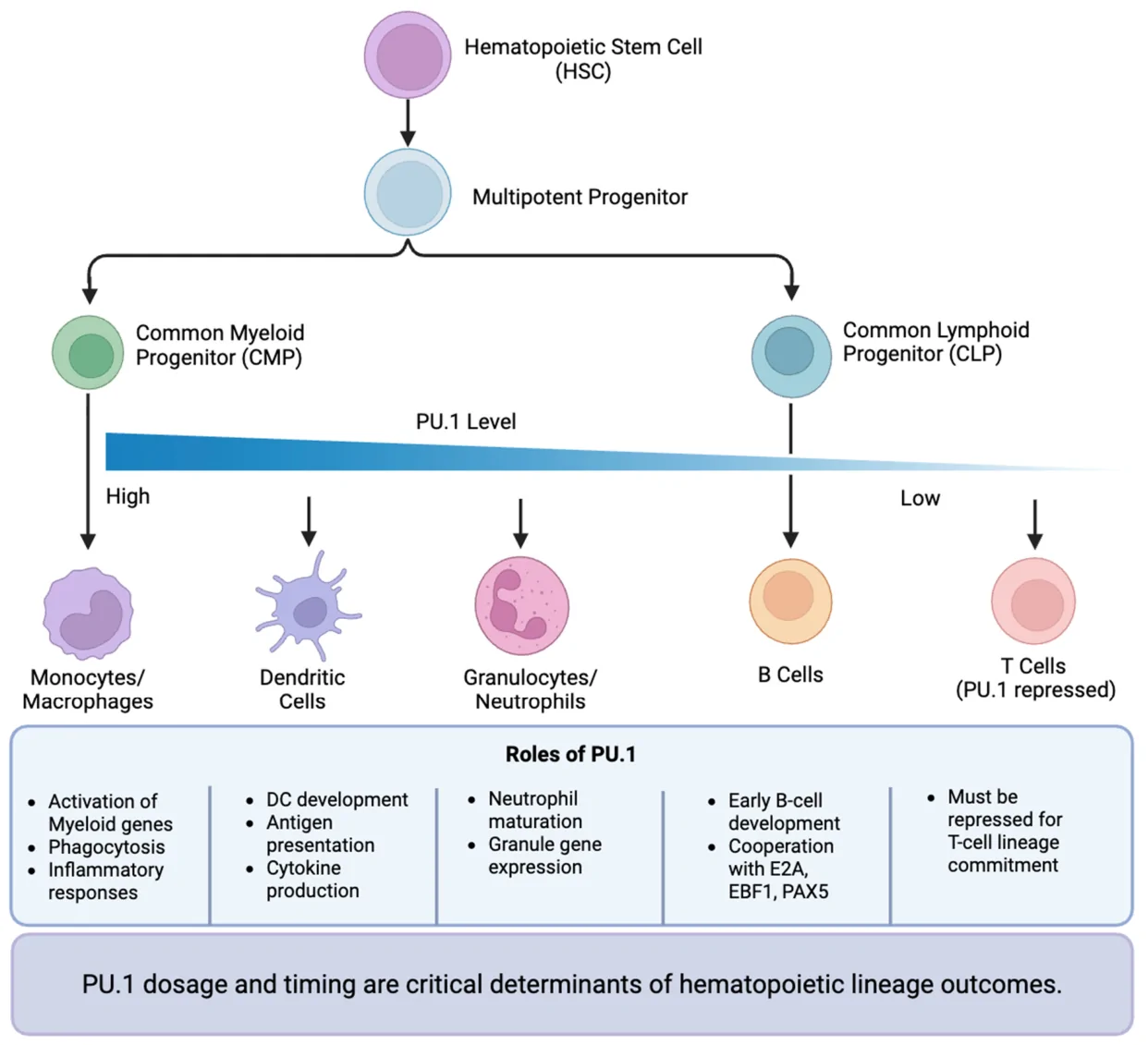
PU.1 in hematopoietic lineage specification. Downstream of HSCs and multipotent progenitors, a concentration gradient of PU.1 dictates lineage specification. High PU.1 levels promote myeloid differentiation from CMPs, driving key functions in monocytes/macrophages, dendritic cells, and neutrophils. Lower concentrations favor lymphoid differentiation from CLPs to support early B-cell development in conjunction with E2A, EBF1, and PAX5, whereas active repression of PU.1 is required for successful T-cell commitment.

The dosage sensitivity of PU.1 in humans is further illustrated by germline PU.1 loss-of-function variants, which cause an autosomal-dominant primary immunodeficiency known as PU.1-mutated agammaglobulinemia (PU.MA). Affected individuals are haploinsufficient for PU.1, presenting with agammaglobulinemia, near-absent circulating B cells, and deficiencies of conventional and plasmacytoid dendritic cells. Disease-causing variants destabilize PU.1, impair its nuclear localization, or disrupt transcriptional activity [104,130]. Notably, in a functional survey of 134 germline variants, including 33 heterozygous loss-of-function carriers, the penetrance of immunodeficiency was approximately 82%, yet none of the carriers developed hematologic malignancy [130]. Thus, constitutional PU.1 haploinsufficiency primarily disrupts humoral and dendritic-cell immunity, contrasting with the somatic, largely non-mutational PU.1 reduction implicated in myeloid transformation (Figure 4).

## 8. PU.1 in Cancer

Given its central roles in lineage specification, differentiation, and chromatin regulation, PU.1 is highly relevant to hematologic malignancy. Altered PU.1 dosage, impaired DNA-binding function, disrupted cofactor interactions, or aberrant epigenetic control can derail normal hematopoiesis and promote transformation. In many contexts, PU.1 acts as a tumor suppressor by enforcing differentiation and restraining self-renewal [131]. However, residual PU.1 activity may also be co-opted by established leukemias to sustain malignant transcriptional programs. Thus, PU.1 can function both as a barrier to transformation and, in specific settings, a dependency of leukemia maintenance [132,133]. Although the tumor-suppressive functions of PU.1 are best characterized in hematologic malignancies, emerging evidence indicates that PU.1 can act as an oncogenic driver when aberrantly overexpressed in certain solid tumors, underscoring its strongly context- and lineage-dependent role in cancer.

### 8.1. Acute Myeloid Leukemia

Reduced *PU.1* expression is one of the most well-established leukemogenic mechanisms in AML. Experimental models show that partial, rather than complete, loss of PU.1 is particularly oncogenic. For instance, mice carrying hypomorphic *PU.1* alleles that markedly reduce *PU.1* expression develop AML-like disease, demonstrating that graded suppression of PU.1 can create a block in differentiation while preserving proliferative capacity [19]. Even modest reductions in PU.1 levels are sufficient to generate myeloid-biased preleukemic stem cells that can progress to AML, in part through inhibition of cooperative transcription factors such as IRF8 [9]. Mechanistically, low PU.1 levels impair activation of monocyte and macrophage differentiation programs, maintain immature blast phenotypes, and disrupt enhancer landscapes required for normal myeloid maturation [134]. In contrast, restoration of wild-type PU.1 in AML model systems increases chromatin accessibility, promotes histone acetylation, activates monocyte/macrophage transcriptional programs, and suppresses leukemic growth by driving terminal differentiation [134,135]. Together, these findings support the concept of PU.1 as a dose-sensitive tumor suppressor in AML.

PU.1 function may also be compromised by mutations, although direct somatic PU.1 mutation is a rare leukemogenic event in human AML. While an early study reported heterozygous PU.1 coding mutations in approximately 7% of AML cases, this frequency was not observed in subsequent cohorts, and dedicated resequencing of the *PU.1* untranslated and promoter regions failed to detect mutations in other AML series [136,137]. Consistent with this, *PU.1* is not among the recurrently mutated driver genes identified in large next-generation sequencing studies of AML [138,139]. Thus, somatic PU.1 coding mutations appear to be an uncommon direct cause of leukemogenesis. The AML-associated PU.1 variants described to date, including mutations within the ETS domain that impair DNA binding, cooperative factor recruitment, or chromatin-opening activity, provide mechanistic evidence that PU.1 dysfunction can contribute to leukemogenesis. Rather than representing a high-frequency driver event, these variants highlight how disruption of PU.1 function can promote leukemic transformation. Instead, PU.1 activity in human AML is more commonly reduced through transcriptional and functional mechanisms, including fusion oncoprotein-mediated repression, enhancer or upstream regulatory element lesions, and microRNA-mediated silencing, as discussed above.

In addition to direct genetic alterations, PU.1 activity is frequently suppressed by leukemia-associated fusion oncoproteins. In core-binding factor leukemias, fusion proteins such as RUNX1::RUNX1T1 [t(8;21)] can physically interact with PU.1 and displace essential cofactors like c-Jun, thereby repressing PU.1 transcriptional activity while leaving its DNA binding partially intact [140]. Moreover, RUNX1::RUNX1T1 suppresses expression of the lncRNA inducer of PU.1, *LOUP* [70]. Consequently, PU.1 target-gene expression is downregulated, resulting in myeloid differentiation arrest [70,140]. Similarly, other fusion proteins, including PML-RARA in acute promyelocytic leukemia, indirectly inhibit PU.1-driven differentiation programs through repressive chromatin mechanisms [141]. These examples highlight how leukemias can disable PU.1 function not only through mutation, but also via dominant transcriptional repression. Conversely, established leukemias can co-opt residual PU.1 activity through aberrantly expressed partner proteins. Paraspeckle component 1 (PSPC1) is overexpressed in AML and associated with poor survival. Although dispensable for normal hematopoiesis, PSPC1 is required to maintain the leukemic state by cooperatively binding chromatin with PU.1 and activating tumor-promoting genes such as *NDC1*. Loss of PSPC1 induces leukemic differentiation and abolishes leukemogenesis [142].

Because RUNX1/CBFβ pathways intersect closely with PU.1 regulation, disruption of core-binding factor complexes can profoundly alter PU.1-dependent maturation [33,92]. RUNX1 normally supports *PU.1* expression and collaborates with PU.1 at myeloid target genes. Loss of RUNX1 function or expression of dominant-negative RUNX1 fusion proteins can shift PU.1-bound loci toward repression, impair coactivator recruitment, and sustain progenitor-like transcriptional states [33,93]. Interestingly, residual RUNX–PU.1 signaling may still be required for leukemia stem cell survival in some AML subtypes. Experimental disruption of RUNX-binding elements within PU.1 enhancers can delay leukemogenesis and reduce leukemia-initiating capacity, highlighting a paradox in which partial pathway activity supports malignant maintenance even though full physiologic activity suppresses transformation [143].

As discussed above, somatic coding mutations of PU.1 are rare, and reduced PU.1 activity in human AML is achieved predominantly through cis-regulatory, epigenetic, signaling, and post-transcriptional mechanisms. Lesions of the −14 kb upstream regulatory element (URE), including heterozygous locus deletion, enhancer SNPs that disrupt SATB1 or NF-κB binding, and DNA or histone methylation of the URE mediated by GATA-1/DNMT1, reduce PU.1 dosage and can be leukemogenic [144,145]. Additional mechanisms, including repression by fusion oncoproteins, aberrant transcription-factor signaling, and microRNA-mediated silencing, further disrupt PU.1 regulation in leukemia [145,146]. Signaling lesions also converge on PU.1: FLT3-ITD suppresses PU.1 directly and indirectly by inducing miR-155 through NF-κB/STAT5 signaling [147], while TP53 loss reduces PU.1 through a MYB/miR-155 axis [148]. Finally, loss of positive regulators also contributes, as C/EBPα directly activates the PU.1 enhancer [149], while RUNX1 mutations that disrupt RUNX1–MLL cooperation reduce activating H3K4me3 at the *PU.1* locus [150].

### 8.2. Myelodysplastic Syndromes

Insufficient PU.1 activity may also contribute to MDS, where ineffective hematopoiesis, dysplasia, and impaired differentiation are central features [9,151,152]. Reduced PU.1 expression or defective upstream regulatory pathways may compromise maturation of myeloid progenitors, leading to cytopenias and abnormal cellular morphology [151,152]. In selected MDS contexts, progressive suppression of PU.1-regulated programs may also facilitate transition toward secondary AML [9]. Although PU.1 alterations are less uniform in MDS than in AML, the broader principle remains consistent: impaired PU.1 function destabilizes normal differentiation and hematopoietic homeostasis.

### 8.3. Lymphoid Malignancies

PU.1 dysregulation is also implicated in B-cell neoplasms. Reduced *PU.1* expression has been observed in diffuse large B-cell lymphoma (DLBCL), particularly in non-germinal center subtypes, consistent with a tumor-suppressive role in mature B cells. Conditional deletion of PU.1 in murine B cells can promote lymphoma formation, and restoration of PU.1 suppresses disease phenotypes in experimental systems [153]. Loss of both PU.1 and the related ETS factor Spi-B has been associated with aggressive pre-B acute lymphoblastic leukemia models [154]. This underscores the importance of ETS-family transcriptional control in B-cell development. In multiple myeloma, PU.1 can be silenced through methylation of upstream regulatory elements, and forced PU.1 re-expression induces growth arrest and apoptosis [155]. Altered PU.1 dosage or DNA-binding specificity has also been linked to Waldenström macroglobulinemia and other mature B-cell malignancies [30].

### 8.4. MicroRNA and Epigenetic Suppression of PU.1

Beyond mutations and translocations, PU.1 can be repressed by noncoding RNA and epigenetic mechanisms. Oncogenic miR-155, frequently elevated in AML and lymphoma, directly suppresses *PU.1* expression [147,156]. Likewise, promoter or enhancer methylation can reduce *PU.1* transcription in lymphoid and plasma-cell malignancies [155,157]. This also applies to myeloid diseases, in which aberrant DNA methylation can disrupt PU.1 regulation. DNA hypermethylation of the −14 kb upstream regulatory element (URE) is associated with reduced *PU.1* expression in CD34+ cells from patients with high-risk MDS, while in AML erythroleukemia, GATA-1 recruits DNMT1 to methylate the URE and suppress *PU.1* expression [98]. Conversely, hypomethylating agents such as azacitidine can relieve aberrant DNA methylation and partially restore *PU.1* expression, providing a mechanistic link between epigenetic dysregulation of PU.1 and the therapeutic activity of DNA methyltransferase inhibition in myeloid malignancies [151,158]. Together, these mechanisms reinforce the concept that impaired PU.1 regulation is a recurrent route to malignant transformation across hematologic cancers. Collectively, hematologic malignancies illustrate the profound dosage sensitivity of PU.1 biology. Too little PU.1 impairs differentiation and promotes malignant transformation, whereas residual PU.1 activity can be co-opted by established cancers. Understanding how PU.1 is dysregulated in specific disease contexts provides a framework for precision therapies aimed at restoring normal lineage control or disrupting malignant transcriptional circuitry.

### 8.5. PU.1 in Solid Tumors

In contrast to its predominantly tumor-suppressive role in the myeloid compartment, *PU.1* is frequently overexpressed and functions as an oncogene in a range of non-hematopoietic cancers. In head and neck squamous cell carcinoma, PU.1 was recently identified as a driver of genome-wide mRNA hypertranscription, a hallmark of oncogenic transformation. Gain- and loss-of-function studies showed that PU.1 alters nascent RNA synthesis and promotes cell proliferation, migration, invasion, and xenograft tumor growth, while high PU.1 activity is associated with activation of oncogenic pathways and poor clinical outcomes [159]. Comparable oncogenic activity has been reported in other epithelial and non-hematopoietic malignancies. PU.1 is upregulated and associated with adverse prognosis in gastric cancer [160]; drives glycolysis and metastasis through HK2 in melanoma [161]; and transcriptionally activates *CST2* to promote proliferation, metastasis, and angiogenesis in esophageal carcinoma [162]. In addition, PU.1 promotes radioresistance in lung squamous cell carcinoma and nasopharyngeal carcinoma [163], and correlates with HER2 status and shorter survival in breast cancer [164]. PU.1 also contributes to the tumor-promoting stroma. In castration-resistant prostate cancer, PU.1 was identified as a driver of the profibrotic phenotype of cancer-associated fibroblasts, and pharmacologic PU.1 inhibition reversed this profibrotic program and reduced tumor organoid growth [165]. Together, these findings extend the relevance of PU.1 beyond hematologic malignancies and suggest that, in selected solid tumors, PU.1 inhibition rather than restoration may be therapeutically desirable.

## 9. PU.1 in Chronic Inflammation, Fibrosis, and Neurodegenerative Disease

Beyond its established roles in hematopoiesis and malignancy, PU.1 is increasingly recognized as a key regulator of chronic inflammatory disorders, tissue remodeling, innate immune memory, and neurodegeneration. Because PU.1 controls macrophage and microglial identity, cytokine responsiveness, enhancer accessibility, and inflammatory gene expression, even modest alterations in PU.1 activity can have profound consequences for systemic and tissue-specific disease states.

### 9.1. Chronic Inflammatory Disease and Autoimmunity

PU.1-regulated macrophage activation contributes to multiple chronic inflammatory disorders, including atherosclerosis, autoimmune diseases, and persistent tissue inflammation [82,166,167]. Macrophages rely on PU.1 to maintain the expression of pattern-recognition receptors, Fc receptors, cytokine receptors, and inflammatory mediators [82,168,169]. Through these pathways, PU.1 helps determine how macrophages respond to microbial products, damaged tissue, lipid accumulation, and immune complexes.

In atherosclerosis, PU.1-dependent macrophage programs may promote foam-cell formation, cytokine production, and plaque inflammation [170,171]. In autoimmune settings, excessive PU.1-driven activation of monocytes, macrophages, and dendritic cells can amplify antigen presentation and pathogenic cytokine networks [116,172]. In several disease models, PU.1 is actively upregulated rather than merely expressed. PU.1 is induced in activated macrophages and in the inflamed CNS during experimental autoimmune encephalomyelitis, in part through loss of the PU.1-targeting miR-150 [173]. In psoriasis, PU.1 drives the expansion and M1 polarization of intermediate monocytes through Dectin-1/SYK/NF-κB signaling [174]. This elevated expression can be sustained by upstream myeloid regulators. For example, C/EBPα induces PU.1 together with the enhancer RNA *LOUP* in a feed-forward circuit that promotes monocyte-to-macrophage differentiation and inflammatory cytokine production [62]. The consequences are nonetheless context-dependent, as PU.1 in synovial resident macrophages can exert protective effects in rheumatoid arthritis. In this setting, PU.1 promotes efferocytosis and restrains NLRP3 activation [175]. Conversely, inadequate PU.1 activity may impair resolution programs and tissue repair [79,176]. Thus, depending on the context, PU.1 appears to regulate both the initiation and persistence of inflammation. Because PU.1 functions upstream of multiple innate immune pathways, it may serve as a convergent regulatory node linking environmental triggers to chronic immune pathology.

### 9.2. Fibrosis and Tissue Remodeling

PU.1 has also been implicated in profibrotic gene expression in fibroblasts and pathological tissue remodeling [177]. Although traditionally viewed as a hematopoietic factor, PU.1-regulated programs in stromal and inflammatory microenvironments may contribute to extracellular matrix deposition, fibroblast activation, and chronic wound-healing responses. In fibrotic diseases, PU.1-dependent cytokines produced by macrophages can further stimulate fibroblast activation [178], suggesting that PU.1 may influence fibrosis both directly and indirectly through immune–stromal crosstalk. The same profibrotic fibroblast program can be co-opted within the tumor microenvironment. In castration-resistant prostate cancer, stromal PU.1 drives a profibrotic cancer-associated fibroblast phenotype that supports tumor growth and can be reversed by PU.1 inhibition, linking PU.1-dependent fibrosis to solid-tumor progression [165]. This may be particularly relevant in the lung, liver, kidney, and cardiovascular systems, where persistent inflammation drives progressive fibrotic remodeling [178,179].

### 9.3. PU.1 in Microglial Biology

Within the central nervous system, PU.1 is highly enriched in microglia and functions as a master regulator of microglial development, identity, survival, and homeostasis. PU.1 controls expression of genes involved in phagocytosis, antigen presentation, cytokine signaling, and tissue surveillance [128,180]. Loss or reduction of PU.1 disrupts core microglial transcriptional programs and impairs cellular viability or function [128,181,182]. PU.1 also cooperates with transcription factors such as IRF8 in positive regulatory loops that reinforce activated microglial states. Composite IRF–ETS motifs are enriched near genes involved in neuroimmune responses, indicating that PU.1 integrates developmental identity with inducible inflammatory signaling in the brain [183].

PU.1 activity in microglia is also regulated by noncoding RNAs. The aging-associated, glia-enriched long noncoding RNA *Glelr* (human homolog, *GLELR*) physically interacts with PU.1 and restrains its inflammatory transcriptional activity. Knockdown of *Glelr* in primary mouse and iPSC-derived human microglia enhances pro-inflammatory cytokine expression, including TNFα, and increases phagocytic activity [184]. These transcriptional changes are enriched for TNF and complement signaling and overlap with PU.1-driven programs. Notably, *GLELR* expression is reduced in postmortem Alzheimer’s disease brains, and Alzheimer’s disease-associated genes are enriched among its targets, positioning the *GLELR*–PU.1 axis as a potential modulator of microglial neuroinflammation in neurodegeneration [184].

Through its regulation of microglial functions, including synaptic pruning, debris clearance, and inflammatory responses, PU.1 plays a critical role in maintaining central nervous system immune homeostasis.

### 9.4. Alzheimer’s Disease and Neurodegeneration

Human genetic studies have linked PU.1-associated myeloid networks to Alzheimer’s disease (AD) risk [22,126]. Variants that reduce *PU.1* expression in myeloid cells have been associated with delayed disease onset, whereas higher *PU.1* expression correlates with increased AD susceptibility [22]. These observations suggest that PU.1 dosage modulates neurodegenerative risk through effects on microglial inflammatory state and phagocytic behavior. PU.1 appears to play a context-dependent dual role in AD. Increased PU.1 activity may enhance pro-inflammatory microglial responses that contribute to neuronal injury, while controlled or reduced *PU.1* expression may favor immunoregulatory states that limit pathology [18,182]. Experimental studies have shown that lowering PU.1 can reduce amyloid plaque burden in some models, whereas PU.1 is also required for certain protective phagocytic programs [180,182]. Thus, both excessive and insufficient PU.1 activity may be harmful depending on disease stage and microglial state. PU.1 directly regulates genes relevant to AD risk and microglial function, including *TREM2*, a major phagocytic receptor implicated in amyloid clearance [126]. Reduction in PU.1 impairs amyloid-β uptake in some settings, emphasizing the need for balanced rather than uniformly suppressed PU.1 activity [128,180].

### 9.5. Other Neurodegenerative Conditions

PU.1 dysfunction may also contribute to disorders beyond AD. In C9ORF72-associated amyotrophic lateral sclerosis/frontotemporal dementia (ALS/FTD), PU.1-centered transcriptional networks have been reported to be suppressed in patient-derived microglia, and restoration of PU.1 can rescue phagocytic and gene-expression defects [185,186]. This raises the possibility that inadequate PU.1 activity may underlie broader forms of microglial failure across neurodegenerative diseases. Collectively, PU.1 connects innate immune identity with chronic inflammation, tissue remodeling, and neurodegeneration. In peripheral tissues, PU.1 shapes macrophage-driven inflammatory and fibrotic responses [82,177]. In the brain, it governs microglial homeostasis and influences susceptibility to Alzheimer’s disease and related disorders. These findings underscore PU.1 as a context-dependent regulator whose precise dosage and chromatin activity determine whether immune responses are protective, maladaptive, or therapeutically reprogrammable.

## 10. Therapeutic Strategies Targeting PU.1

**Figure 5:**
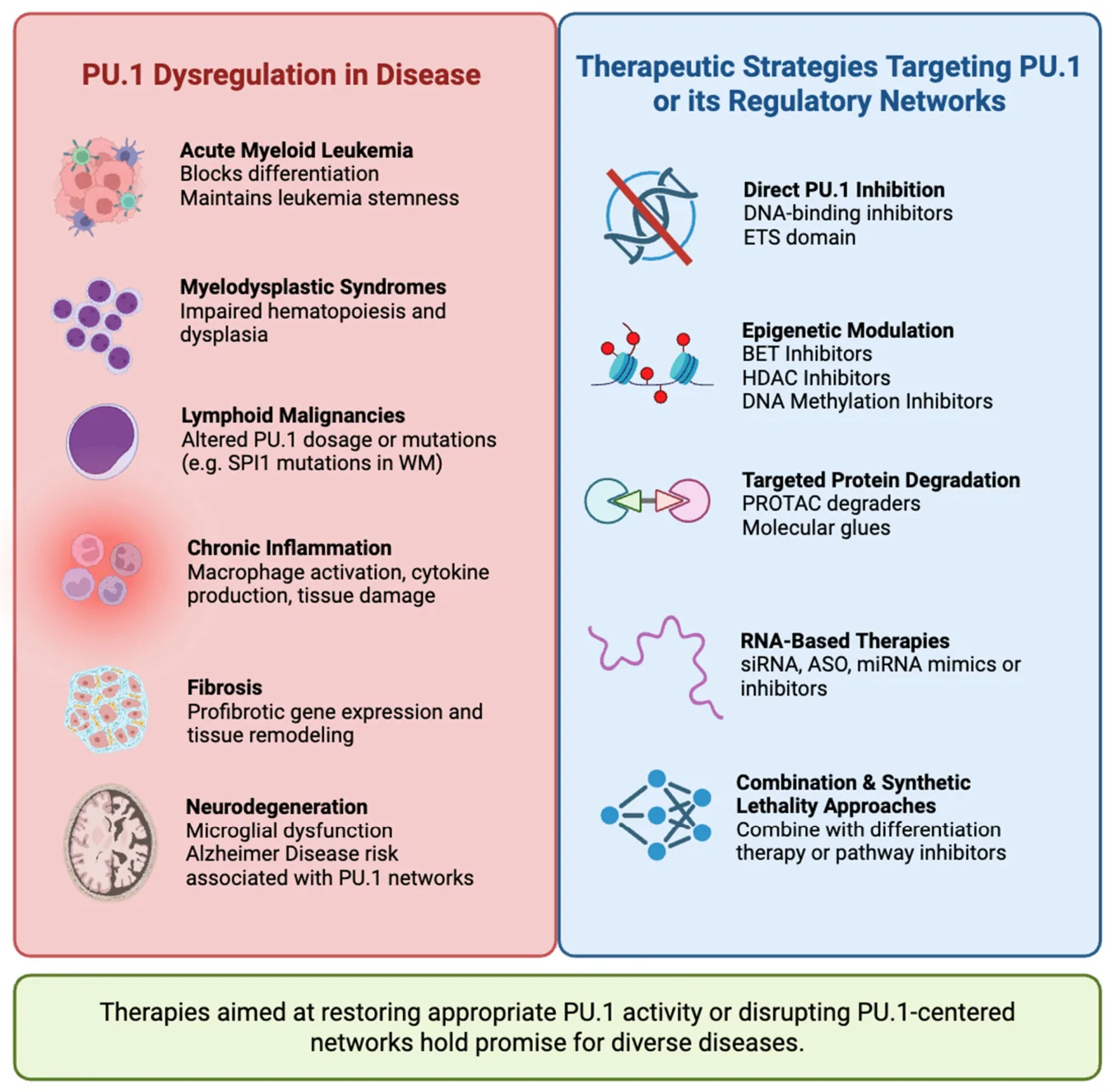
PU.1 in disease and relevant therapeutic strategies. An overview of diseases associated with PU.1 dysfunction and the corresponding therapeutic interventions designed to restore homeostatic activity. PU.1 dysregulation contributes to acute myeloid leukemia (AML), myelodysplastic syndromes, lymphoid malignancies, chronic inflammatory tissue damage, fibrosis, and microglial-mediated neurodegeneration in Alzheimer’s disease. Current and emerging therapeutic approaches target these networks via direct ETS-domain DNA-binding inhibitors, epigenetic modifiers, PROTAC- or molecular glue-mediated targeted protein degradation, RNA-based interference (siRNA/ASO), and synthetic lethality combinations (Table 2).

Given its central role in hematopoietic differentiation, immune activation, chromatin remodeling, and disease pathogenesis, PU.1 has emerged as an attractive but complex therapeutic target. Because PU.1 can function as either a tumor suppressor or a disease-promoting factor depending on cellular context, optimal therapeutic strategies must be tailored to the underlying pathology. In hematologic malignancies such as AML, where PU.1 activity is frequently reduced, restoring PU.1 function may promote differentiation and suppress leukemic self-renewal [135] (Figure 5). In contrast, in chronic inflammatory and neurodegenerative disorders, excessive or maladaptive PU.1-driven myeloid activation may justify partial inhibition or reprogramming of PU.1-dependent transcriptional networks [129,187] (Figure 5).

**Table 2:** Therapeutic approaches targeting PU.1.

Strategy	Mechanism	Development Status
DNA-binding inhibitors	Block ETS motif engagement or alter chromatin occupancy	Preclinical
BET inhibitors	Suppress PU.1-driven enhancer activity	Clinical for AML/MDS, not PU.1 targeted
HDAC inhibitors	Reprogram chromatin and indirectly modulate PU.1 outputs	Clinical use in subsets
LSD1 inhibitors	Recommission PU.1/C/EBPα enhancers and induce differentiation	Clinical for AML/MPN, not PU.1 targeted
PROTAC degraders	Targeted PU.1 protein degradation	Emerging concept
RNA therapeutics	siRNA/ASO targeting *PU.1* transcripts	Experimental
Synthetic lethality	Target PU.1 cofactor or chromatin dependencies	Active research

### 10.1. Direct Inhibition of PU.1 Activity

First-in-class small molecules targeting PU.1 have demonstrated proof of concept that transcription factors can be pharmacologically modulated. Heterocyclic diamidines such as DB1976 and DB2313 bind the DNA minor groove adjacent to PU.1 recognition motifs, thereby interfering with PU.1-chromatin interactions and suppressing canonical PU.1 target gene expression [188]. In AML models, these compounds reduce leukemic growth, impair clonogenicity, and prolong survival in xenograft systems [188]. Additional DNA-targeting polyamides can redistribute PU.1 binding across the genome, in some cases paradoxically inducing differentiation through activation of alternate transcriptional programs [189]. The therapeutic reach of these agents may extend beyond hematopoietic malignancy. As noted above, pharmacologic PU.1 inhibition reverses the profibrotic cancer-associated fibroblast phenotype and reduces tumor organoid growth in castration-resistant prostate cancer [165], supporting stromal PU.1 as druggable nodes in solid tumors.

### 10.2. Restoration of PU.1 Function in Leukemia

Because many leukemias depend on differentiation arrest caused by reduced PU.1 activity, restoration of PU.1 function represents a rational therapeutic strategy. Experimental overexpression of PU.1 in AML blasts induces myelomonocytic differentiation, reduces proliferation, and increases apoptosis [135]. Similar effects have been observed in RUNX1::RUNX1T1–positive leukemias, where *PU.1* re-expression can overcome fusion protein-mediated repression [140]. Future therapeutic strategies may include reactivating PU.1 transcription, reversing enhancer methylation, inhibiting PU.1-targeting microRNAs, or disrupting oncogenic complexes that suppress PU.1 activity.

### 10.3. Epigenetic Modulation of PU.1 Networks

Because PU.1 acts largely through enhancer circuits, epigenetic therapies may indirectly reprogram PU.1-dependent transcription. BET inhibitors suppress super-enhancer activity and may attenuate pathogenic PU.1-driven programs in inflammatory states and cancer [190]. HDAC inhibitors can alter chromatin accessibility and have been shown to reduce *PU.1* expression in microglia [129]. Hypomethylating agents such as 5-azacitidine may restore *PU.1* expression in myelodysplastic syndromes by reversing silencing of upstream regulatory elements [151]. LSD1 inhibition has also shown promise by reactivating PU.1- and C/EBPα-dependent enhancers, thereby promoting leukemic differentiation [191].

### 10.4. Targeting PU.1 Cofactors and Dependencies

An alternative strategy is to exploit synthetic lethal dependencies within PU.1 regulatory networks. In AML, PU.1 cooperates with chromatin remodelers such as SWI/SNF complexes to maintain oncogenic transcriptional states. Pharmacologic disruption of SWI/SNF function reduces chromatin accessibility at PU.1-bound enhancers and promotes leukemic differentiation [76]. Similarly, inhibition of signaling pathways that destabilize PU.1 protein, such as GSK3β-mediated degradation pathways [45], can restore PU.1 levels and re-engage myeloid maturation programs.

### 10.5. RNA Therapeutics and Protein Degradation

Next-generation approaches include antisense oligonucleotides (ASOs), siRNA therapeutics, and PROTAC-based degraders. RNA-directed therapies could selectively reduce *PU.1* expression in diseases characterized by excessive PU.1 activity, while preserving broader transcriptional networks [187]. PROTAC strategies remain early-stage but could provide a means to degrade PU.1 or specific PU.1-containing oncogenic complexes with greater precision than DNA-binding inhibitors. Analogously, targeting the deubiquitinase USP7, which stabilizes PU.1 by preventing its proteasomal degradation [47], could provide an indirect strategy to reduce PU.1 levels in contexts of pathological PU.1 activity.

### 10.6. PU.1 in Neurodegeneration and Microglial Reprogramming

In Alzheimer’s disease and related neurodegenerative disorders, higher *PU.1* expression has been associated with increased disease risk [22], suggesting that partial attenuation of PU.1 may reduce harmful neuroinflammatory responses. Experimental knockdown of PU.1 in microglia decreases pro-inflammatory gene expression while preserving some phagocytic capacity [18]. However, PU.1 is also required for beneficial microglial transitions induced by IL-33 and other reparative signals [192], suggesting that complete inhibition may be detrimental. Accordingly, therapeutic reprogramming rather than simple suppression of PU.1 may offer the most effective strategy in the central nervous system.

### 10.7. Challenges and Future Directions

Despite growing interest, therapeutic targeting of PU.1 presents significant challenges. PU.1 regulates multiple immune lineages, and systemic inhibition could impair host defense or normal hematopoiesis. Conversely, broad activation may promote inflammatory pathology. Achieving lineage-specific, dose-sensitive, and temporally controlled modulation of PU.1 will therefore be essential.

A central obstacle to directly targeting PU.1 is selectively delivering therapeutic agents to the relevant cell populations while sparing other lineages. Several approaches could be explored to address this challenge and enable cell type-specific modulation of PU.1. Antibody- and ligand-targeted lipid nanoparticles (LNPs) can restrict nucleic-acid delivery to defined cell populations. For example, c-Kit (CD117)-targeted LNPs deliver RNA to hematopoietic stem and progenitor cells in vivo without conditioning [193]. β-integrin-targeted LNPs transfect leukocytes, and hyaluronic acid-functionalized LNPs target CD44-high activated macrophages [194,195]. Off-target myeloid and hepatic uptake can be reduced through CD47 “don’t-eat-me” surface cloaking, while bone marrow-directed formulations are being developed to target leukemic and stem-cell compartments [196,197]. For the central nervous system, blood–brain barrier-penetrant, microglia-selective carriers—including Angiopep-2-decorated nanoparticles [198], detachable albumin-shell gene-delivery systems [199], and engineered AAV capsids with microglia-specific promoters [200]—could offer potential strategies to reprogram PU.1-dependent microglial programs. Finally, targeted PROTAC delivery using leukemia-selective penetrating peptides [201], aptamer/antibody conjugates and cell type-specific E3 ligases [202] may provide the tumor selectivity needed for degrader-based approaches.

Overall, PU.1 represents a highly promising, yet context-dependent therapeutic node. In malignancy, restoring or redirecting PU.1 activity may overcome differentiation arrest. This context dependence extends across cancer types, as leukemias may benefit from PU.1 restoration or redirection, whereas solid tumors may require its selective inhibition. In inflammatory and neurodegenerative diseases, selective dampening or reprogramming of PU.1-dependent myeloid states may prove beneficial.

## 11. Conclusions 

PU.1 has emerged as a master regulator of immune cell identity whose influence extends from early hematopoietic development to mature immune function and disease pathogenesis. As a lineage-defining ETS family transcription factor, PU.1 governs hematopoietic stem and progenitor cell fate decisions, directs differentiation of myeloid and lymphoid populations, and maintains specialized functions in mature immune cells. Its importance is underscored by the fact that relatively small quantitative changes in PU.1 expression can profoundly alter lineage outcomes, highlighting PU.1 as a dosage-sensitive regulator of cellular identity rather than a simple binary transcriptional switch.

Mechanistically, PU.1 functions through multiple integrated layers of regulation. Its modular protein structure enables sequence-specific DNA recognition, recruitment of transcriptional cofactors, and responsiveness to intracellular signaling pathways. Beyond conventional promoter binding, PU.1 acts as a pioneer transcription factor capable of engaging partially inaccessible chromatin, recruiting chromatin remodeling complexes, and establishing lineage-specific enhancer landscapes. Through cooperation with factors such as C/EBP proteins, IRFs, RUNX1, AP-1, and GATA family members, PU.1 orchestrates complex gene regulatory networks that couple intrinsic developmental programs with extracellular environmental cues. These properties position PU.1 not merely as a transcription factor, but as a higher-order regulator of chromatin accessibility, enhancer logic, and immune cell plasticity.

The biological significance of PU.1 is further reflected in the wide spectrum of diseases associated with its dysregulation. In hematologic malignancies, particularly acute myeloid leukemia and myelodysplastic syndromes, reduced or altered PU.1 activity contributes to differentiation arrest and malignant transformation. Beyond the hematopoietic system, PU.1 has increasingly been implicated as an oncogenic driver in solid tumors, including head and neck squamous cell carcinoma, further broadening the therapeutic considerations for targeting this factor. In chronic inflammatory diseases, PU.1-driven macrophage activation programs can exacerbate tissue injury, fibrosis, and atherosclerosis. In the central nervous system, PU.1 is indispensable for microglial identity and function, while genetic and functional studies have linked altered *PU.1* activity to Alzheimer’s disease and other neurodegenerative disorders. Thus, PU.1 lies at the intersection of hematology, immunology, and neurobiology.

Long considered difficult to drug, PU.1 is increasingly becoming a realistic translational target. Advances in structural biology, chromatin therapeutics, targeted protein degradation, RNA-based therapies, and synthetic lethal approaches have created new opportunities to modulate PU.1 activity directly or indirectly. Importantly, therapeutic strategies will need to be disease-specific: restoring PU.1 activity may be beneficial in leukemias characterized by PU.1 insufficiency, whereas selective attenuation or reprogramming of PU.1-dependent myeloid states may be preferable in inflammatory and neurodegenerative conditions. Precision targeting of PU.1 networks, rather than indiscriminate inhibition, will likely define the most successful future interventions.

## 12. Perspective

Looking ahead, several major questions remain unresolved. How PU.1 dosage is quantitatively interpreted during lineage commitment, how aging and chronic inflammation reshape PU.1 cistromes, and which cofactors distinguish normal from pathogenic PU.1 activity are central issues for future study. Additional work is also needed to define noncanonical functions of PU.1 outside traditional hematopoietic contexts and to determine whether PU.1 can be safely modulated without compromising host defense. Overall, PU.1 represents both a foundational model for understanding transcription factor control of cell fate and an increasingly credible therapeutic target. Continued integration of genomic, structural, single-cell, and translational approaches is likely to establish PU.1-centered regulatory circuits as a major frontier in precision immunology, hematology, and neuroimmune medicine.

A major gap remains in identifying which cofactors and chromatin dependencies are uniquely required in leukemia compared with normal hematopoiesis, particularly those that could explain malignant rewiring of PU.1-dependent regulatory networks. This raises an important translational challenge: whether PU.1 can be selectively targeted for therapeutic purposes without compromising essential antimicrobial host defense functions. In parallel, there is growing interest in uncovering potential noncanonical roles of PU.1 beyond hematopoietic and myeloid lineages, which may broaden its biological significance. Finally, early evidence that caloric restriction remodels PU.1 occupancy genome-wide in HSCs illustrates that PU.1 cistrome plasticity is experimentally tractable. It still remains unclear the extent to which aging, chronic inflammation, and environmental stress collectively reshape PU.1 cistromes over time, potentially altering transcriptional programs and contributing to disease susceptibility.

## Figures and Tables

**Table 1:** Major PU.1 binding partners.

Partner	Major Context	Functional Outcome
C/EBPα	Myeloid differentiation	Macrophage/granulocyte enhancers
C/EBPβ	Emergency myelopoiesis	Inflammatory activation
IRF8	Monocyte/DC fate	Antigen presentation programs
IRF4	B cells/DCs	Adaptive immune regulation
RUNX1	Hematopoiesis	Stem/progenitor enhancer control
GATA1	Antagonistic	Erythroid vs. myeloid fate
CBP/p300	Coactivator	Histone acetylation
BRG1/SWI-SNF	Chromatin remodeling	Enhancer opening

## Data Availability

No new data were created or analyzed in this study. Data sharing is not applicable to this article.

## Abbreviations

The following abbreviations are used in this manuscript:

HSC	Hematopoietic stem cell
SPI1	Spi-1 proto-oncogene
ETS	Erythroblast transformation specific
DNA	Deoxyribonucleic acid
C/EBP	CCAAT/enhancer-binding protein
IRF	Interferon regulatory factor
RUNX1	Runt-related transcription factor 1
MDS	Myelodysplastic syndrome
TAD	Transactivation domain
PEST	Proline/glutamate/serine/threonine
GATA	GATA-binding proteins
CBP	CREB-binding protein
TFIID	Transcription factor IID
SWI/SNF	SWItch/Sucrose non-fermentable
CSF1R	Macrophage colony-stimulating factor 1 receptor
RNA	Ribonucleic acid
mRNA	Messenger ribonucleic acid
UTR	Untranslated regions
miR	Micro ribonucleic acid
AKT	RAC-alpha serine/threonine protein kinase
HDAC	Histone deacetylase
DNMT	DNA Methyltransferase
CSF2RA	Colony-stimulating factor 2 receptor subunit alpha
CSF3R	Colony-stimulating factor 3 receptor
LDB1	LIM Domain Binding 1
lncRNA	Long-noncoding ribonucleic acid
eRNA	Enhancer ribonucleic acid
*LOUP*	lncRNA originating from upstream regulatory element of PU.1
ATP	Adenosine triphosphate
H3K4me1	Histone H3 lysine 4 monomethylation
H3K27ac	Histone H3 lysine 27 acetylation
NF-κB	Nuclear factor kappa B
AP-1	Activator protein 1
PRC2	Polycomb repressive complex 2
H3K27me3	Histone H3 lysine 27 trimethylation
IL1B	Interleukin 1 beta
subTAD	Sub-topologically associating domain
BRG1	Brahma-related gene 1
CMP	Common myeloid progenitor
GMP	Granulocyte–monocyte progenitor
CLP	Common lymphoid progenitor
DC	Dendritic cell
STAT	Signal transducer and activator of transcription
TREM2	Triggering receptor expression on myeloid cells 2
EBF1	Early B-cell factor 1
PAX5	Paired box protein Pax-5
RUNX1::RUNX1T1	Runt-related transcription factor 1 fused with RUNX1 Partner Transcriptional Co-Repressor 1
PML	Promyelocytic leukemia
RARA	Retinoic acid receptor alpha
DLBCL	Diffuse large B-cell lymphoma
AD	Alzheimer’s disease
C9ORF72	Chromosome 9 open reading frame 72
ALS	Amyotrophic lateral sclerosis
FTD	Frontotemporal dementia
BET	Bromodomain and extra-terminal motif
LSD1	Lysine-specific demethylase 1
GSK3β	Glycogen synthase kinase 3 beta
ASO	Antisense oligonucleotide
siRNA	Small interfering ribonucleic acid
PROTAC	Proteolysis targeting chimera
IL-33	Interleukin 33
WM	Waldenström macroglobulinemia

## References

[1] Rosenbauer F., Tenen D.G. (2007). Transcription factors in myeloid development: Balancing differentiation with transformation. Nat. Rev. Immunol..

[2] Orkin S.H., Zon L.I. (2008). Hematopoiesis: An evolving paradigm for stem cell biology. Cell.

[3] Laurenti E., Gottgens B. (2018). From haematopoietic stem cells to complex differentiation landscapes. Nature.

[4] McKercher S.R., Torbett B.E., Anderson K.L., Henkel G.W., Vestal D.J., Baribault H., Klemsz M., Feeney A.J., Wu G.E., Paige C.J. (1996). Targeted disruption of the *PU.1* gene results in multiple hematopoietic abnormalities. EMBO J..

[5] Korczmar E.A., Bookstaver A.K., Ober E., Goldfarb A.N., Tenen D.G., Trinh B.Q. (2024). Transcriptional Regulation of the Lineage-Determining Gene *PU.1* in Normal and Malignant Hematopoiesis: Current Understanding and Therapeutic Perspective. Front. Biosci.-Sch..

[6] Scott E.W., Simon M.C., Anastasi J., Singh H. (1994). Requirement of transcription factor PU.1 in the development of multiple hematopoietic lineages. Science.

[7] Nutt S.L., Metcalf D., D’Amico A., Polli M., Wu L. (2005). Dynamic regulation of *PU.1* expression in multipotent hematopoietic progenitors. J. Exp. Med..

[8] Li G., Hao W., Hu W. (2020). Transcription factor PU.1 and immune cell differentiation. Int. J. Mol. Med..

[9] Will B., Vogler T.O., Narayanagari S., Bartholdy B., Todorova T.I., da Silva Ferreira M., Chen J., Yu Y., Mayer J., Barreyro L. (2015). Minimal PU.1 reduction induces a preleukemic state and promotes development of acute myeloid leukemia. Nat. Med..

[10] DeKoter R.P., Singh H. (2000). Regulation of B lymphocyte and macrophage development by graded expression of *PU.1*. Science.

[11] Qiu K., Vu D.C., Wang L., Nguyen N.N., Bookstaver A.K., Sol-Church K., Li H., Dinh T.N., Goldfarb A.N., Tenen D.G. (2024). Chromatin structure and 3D architecture define the differential functions of PU.1 regulatory elements in blood cell lineages. Epigenet. Chromatin.

[12] Minderjahn J., Schmidt A., Fuchs A., Schill R., Raithel J., Babina M., Schmidl C., Gebhard C., Schmidhofer S., Mendes K. (2020). Mechanisms governing the pioneering and redistribution capabilities of the non-classical pioneer PU.1. Nat. Commun..

[13] Frederick M.A., Williamson K.E., Fernandez Garcia M., Ferretti M.B., McCarthy R.L., Donahue G., Luzete Monteiro E., Takenaka N., Reynaga J., Kadoch C. (2023). A pioneer factor locally opens compacted chromatin to enable targeted ATP-dependent nucleosome remodeling. Nat. Struct. Mol. Biol..

[14] Heinz S., Benner C., Spann N., Bertolino E., Lin Y.C., Laslo P., Cheng J.X., Murre C., Singh H., Glass C.K. (2010). Simple combinations of lineage-determining transcription factors prime cis-regulatory elements required for macrophage and B cell identities. Mol. Cell.

[15] Ghisletti S., Barozzi I., Mietton F., Polletti S., De Santa F., Venturini E., Gregory L., Lonie L., Chew A., Wei C.L. (2010). Identification and characterization of enhancers controlling the inflammatory gene expression program in macrophages. Immunity.

[16] Jin F., Li Y., Ren B., Natarajan R. (2011). PU.1 and C/EBP(alpha) synergistically program distinct response to NF-kappaB activation through establishing monocyte specific enhancers. Proc. Natl. Acad. Sci. USA.

[17] Vallelonga V., Gandolfi F., Zampini M., Riva E., Maggioni G., Ventura D., Saba E., Termanini A., Polletti S., Prosperini E. (2026). PU.1-Activated Genomic Regions Define Low-risk MDS Subsets Characterized by Immune Dysregulation and Disease Progression. Blood.

[18] Pimenova A.A., Herbinet M., Gupta I., Machlovi S.I., Bowles K.R., Marcora E., Goate A.M. (2021). Alzheimer’s-associated *PU.1* expression levels regulate microglial inflammatory response. Neurobiol. Dis..

[19] Rosenbauer F., Wagner K., Kutok J.L., Iwasaki H., Le Beau M.M., Okuno Y., Akashi K., Fiering S., Tenen D.G. (2004). Acute myeloid leukemia induced by graded reduction of a lineage-specific transcription factor, PU.1. Nat. Genet..

[20] Walton M.R., Gibbons H., MacGibbon G.A., Sirimanne E., Saura J., Gluckman P.D., Dragunow M. (2000). *PU.1* expression in microglia. J. Neuroimmunol..

[21] Kierdorf K., Erny D., Goldmann T., Sander V., Schulz C., Perdiguero E.G., Wieghofer P., Heinrich A., Riemke P., Holscher C. (2013). Microglia emerge from erythromyeloid precursors via Pu.1- and Irf8-dependent pathways. Nat. Neurosci..

[22] Huang K.L., Marcora E., Pimenova A.A., Di Narzo A.F., Kapoor M., Jin S.C., Harari O., Bertelsen S., Fairfax B.P., Czajkowski J. (2017). A common haplotype lowers *PU.1* expression in myeloid cells and delays onset of Alzheimer’s disease. Nat. Neurosci..

[23] Kodandapani R., Pio F., Ni C.Z., Piccialli G., Klemsz M., McKercher S., Maki R.A., Ely K.R. (1996). A new pattern for helix-turn-helix recognition revealed by the PU.1 ETS-domain-DNA complex. Nature.

[24] Wei G.H., Badis G., Berger M.F., Kivioja T., Palin K., Enge M., Bonke M., Jolma A., Varjosalo M., Gehrke A.R. (2010). Genome-wide analysis of ETS-family DNA-binding in vitro and in vivo. EMBO J..

[25] Pham T.H., Minderjahn J., Schmidl C., Hoffmeister H., Schmidhofer S., Chen W., Langst G., Benner C., Rehli M. (2013). Mechanisms of in vivo binding site selection of the hematopoietic master transcription factor PU.1. Nucleic Acids Res..

[26] Pio F., Ni C.Z., Mitchell R.S., Knight J., McKercher S., Klemsz M., Lombardo A., Maki R.A., Ely K.R. (1995). Co-crystallization of an ETS domain (PU.1) in complex with DNA. Engineering the length of both protein and oligonucleotide. J. Biol. Chem..

[27] Terrell J.R., Taylor S.J., Schneider A.L., Lu Y., Vernon T.N., Xhani S., Gumpper R.H., Luo M., Wilson W.D., Steidl U. (2023). DNA selection by the master transcription factor PU.1. Cell Rep..

[28] Ungerback J., Hosokawa H., Wang X., Strid T., Williams B.A., Sigvardsson M., Rothenberg E.V. (2018). Pioneering, chromatin remodeling, and epigenetic constraint in early T-cell gene regulation by SPI1 (PU.1). Genome Res..

[29] Poon G.M. (2012). DNA binding regulates the self-association of the ETS domain of PU.1 in a sequence-dependent manner. Biochemistry.

[30] Roos-Weil D., Decaudin C., Armand M., Della-Valle V., Diop M.K., Ghamlouch H., Ropars V., Herate C., Lara D., Durot E. (2019). A Recurrent Activating Missense Mutation in Waldenstrom Macroglobulinemia Affects the DNA Binding of the ETS Transcription Factor SPI1 and Enhances Proliferation. Cancer Discov..

[31] Behre G., Whitmarsh A.J., Coghlan M.P., Hoang T., Carpenter C.L., Zhang D.E., Davis R.J., Tenen D.G. (1999). C-Jun is a JNK-independent coactivator of the PU.1 transcription factor. J. Biol. Chem..

[32] Zhang P., Behre G., Pan J., Iwama A., Wara-Aswapati N., Radomska H.S., Auron P.E., Tenen D.G., Sun Z. (1999). Negative cross-talk between hematopoietic regulators: GATA proteins repress PU.1. Proc. Natl. Acad. Sci. USA.

[33] Hu Z., Gu X., Baraoidan K., Ibanez V., Sharma A., Kadkol S., Munker R., Ackerman S., Nucifora G., Saunthararajah Y. (2011). RUNX1 regulates corepressor interactions of PU.1. Blood.

[34] Reddy V.A., Iwama A., Iotzova G., Schulz M., Elsasser A., Vangala R.K., Tenen D.G., Hiddemann W., Behre G. (2002). Granulocyte inducer C/EBPalpha inactivates the myeloid master regulator PU.1: Possible role in lineage commitment decisions. Blood.

[35] Listman J.A., Wara-aswapati N., Race J.E., Blystone L.W., Walker-Kopp N., Yang Z., Auron P.E. (2005). Conserved ETS domain arginines mediate DNA binding, nuclear localization, and a novel mode of bZIP interaction. J. Biol. Chem..

[36] Escalante C.R., Brass A.L., Pongubala J.M., Shatova E., Shen L., Singh H., Aggarwal A.K. (2002). Crystal structure of PU.1/IRF-4/DNA ternary complex. Mol. Cell.

[37] Yamamoto H., Kihara-Negishi F., Yamada T., Hashimoto Y., Oikawa T. (1999). Physical and functional interactions between the transcription factor PU.1 and the coactivator CBP. Oncogene.

[38] Hagemeier C., Bannister A.J., Cook A., Kouzarides T. (1993). The activation domain of transcription factor PU.1 binds the retinoblastoma (RB) protein and the transcription factor TFIID in vitro: RB shows sequence similarity to TFIID and TFIIB. Proc. Natl. Acad. Sci. USA.

[39] Ha S.D., Cho W., DeKoter R.P., Kim S.O. (2019). The transcription factor PU.1 mediates enhancer-promoter looping that is required for *IL-1beta* eRNA and mRNA transcription in mouse melanoma and macrophage cell lines. J. Biol. Chem..

[40] Pongubala J.M., Van Beveren C., Nagulapalli S., Klemsz M.J., McKercher S.R., Maki R.A., Atchison M.L. (1993). Effect of PU.1 phosphorylation on interaction with NF-EM5 and transcriptional activation. Science.

[41] Lodie T.A., Savedra R., Golenbock D.T., Van Beveren C.P., Maki R.A., Fenton M.J. (1997). Stimulation of macrophages by lipopolysaccharide alters the phosphorylation state, conformation, and function of PU.1 via activation of casein kinase II. J. Immunol..

[42] Celada A., Borras F.E., Soler C., Lloberas J., Klemsz M., van Beveren C., McKercher S., Maki R.A. (1996). The transcription factor PU.1 is involved in macrophage proliferation. J. Exp. Med..

[43] Zhang D.E., Hetherington C.J., Chen H.M., Tenen D.G. (1994). The macrophage transcription factor PU.1 directs tissue-specific expression of the macrophage colony-stimulating factor receptor. Mol. Cell. Biol..

[44] Rogers S., Wells R., Rechsteiner M. (1986). Amino acid sequences common to rapidly degraded proteins: The PEST hypothesis. Science.

[45] Mishra M., Thacker G., Sharma A., Singh A.K., Upadhyay V., Sanyal S., Verma S.P., Tripathi A.K., Bhatt M.L.B., Trivedi A.K. (2021). FBW7 Inhibits Myeloid Differentiation in Acute Myeloid Leukemia via GSK3-Dependent Ubiquitination of PU.1. Mol. Cancer Res..

[46] Konishi Y., Tominaga A. (2006). PU.1 is degraded in differentiation of erythrocytes through a proteasome-dependent pathway. DNA Cell Biol..

[47] Shan H., Zhang Y., Xiao X., Wu W., Wang Y., Zhu C., Bai W., Zhang Z., Zhai Y., Yang L. (2026). USP7 sustains hematopoietic stem cell homeostasis partially via PU.1 stabilization. Int. J. Biol. Sci..

[48] Brass A.L., Zhu A.Q., Singh H. (1999). Assembly requirements of PU.1-Pip (IRF-4) activator complexes: Inhibiting function in vivo using fused dimers. EMBO J..

[49] Nishiyama C., Nishiyama M., Ito T., Masaki S., Masuoka N., Yamane H., Kitamura T., Ogawa H., Okumura K. (2004). Functional analysis of PU.1 domains in monocyte-specific gene regulation. FEBS Lett..

[50] Martinez-Nunez R.T., Louafi F., Friedmann P.S., Sanchez-Elsner T. (2009). MicroRNA-155 modulates the pathogen binding ability of dendritic cells (DCs) by down-regulation of DC-specific intercellular adhesion molecule-3 grabbing non-integrin (DC-SIGN). J. Biol. Chem..

[51] Lu D., Nakagawa R., Lazzaro S., Staudacher P., Abreu-Goodger C., Henley T., Boiani S., Leyland R., Galloway A., Andrews S. (2014). The miR-155-PU.1 axis acts on Pax5 to enable efficient terminal B cell differentiation. J. Exp. Med..

[52] Fukao T., Fukuda Y., Kiga K., Sharif J., Hino K., Enomoto Y., Kawamura A., Nakamura K., Takeuchi T., Tanabe M. (2007). An evolutionarily conserved mechanism for microRNA-223 expression revealed by microRNA gene profiling. Cell.

[53] Solomon L.A., Podder S., He J., Jackson-Chornenki N.L., Gibson K., Ziliotto R.G., Rhee J., DeKoter R.P. (2017). Coordination of Myeloid Differentiation with Reduced Cell Cycle Progression by PU.1 Induction of MicroRNAs Targeting Cell Cycle Regulators and Lipid Anabolism. Mol. Cell. Biol..

[54] Kong K.Y., Owens K.S., Rogers J.H., Mullenix J., Velu C.S., Grimes H.L., Dahl R. (2010). MIR-23A microRNA cluster inhibits B-cell development. Exp. Hematol..

[55] Ma X., Li Y., Sun W., Lou J., Zhang Y., Tan Y., Wang X., Wang K. (2025). Super-enhancer-associated long noncoding RNA lnc-SPI1U mediates SPI1 feedback regulation by interacting with HNRNPH1 and HNRNPF. Oncogene.

[56] Hensold J.O., Stratton C.A., Barth D. (1997). The conserved 5′-untranslated leader of *Spi-1* (PU.1) mRNA is highly structured and potently inhibits translation in vitro but not in vivo. Nucleic Acids Res..

[57] Rieske P., Pongubala J.M. (2001). AKT induces transcriptional activity of PU.1 through phosphorylation-mediated modifications within its transactivation domain. J. Biol. Chem..

[58] Bai Y., Srinivasan L., Perkins L., Atchison M.L. (2005). Protein acetylation regulates both PU.1 transactivation and Ig kappa 3’ enhancer activity. J. Immunol..

[59] Kihara-Negishi F., Suzuki M., Yamada T., Sakurai T., Oikawa T. (2005). Impaired repressor activity and biological functions of PU.1 in MEL cells induced by mutations in the acetylation motifs within the ETS domain. Biochem. Biophys. Res. Commun..

[60] Ghani S., Riemke P., Schonheit J., Lenze D., Stumm J., Hoogenkamp M., Lagendijk A., Heinz S., Bonifer C., Bakkers J. (2011). Macrophage development from HSCs requires PU.1-coordinated microRNA expression. Blood.

[61] Leddin M., Perrod C., Hoogenkamp M., Ghani S., Assi S., Heinz S., Wilson N.K., Follows G., Schonheit J., Vockentanz L. (2011). Two distinct auto-regulatory loops operate at the PU.1 locus in B cells and myeloid cells. Blood.

[62] Waliullah A.S.M., Nguyen T.A., Dziegielewska B., Zhang J., Qiu K., Tran M.L., Nguyen N.N., Wang L., Pan A., Nong M.K. (2026). The C/EBPalpha-(PU.1-LOUP) regulatory circuit regulates monocyte/macrophage development and immune functions. Blood.

[63] Anderson K.L., Smith K.A., Perkin H., Hermanson G., Anderson C.G., Jolly D.J., Maki R.A., Torbett B.E. (1999). PU.1 and the granulocyte- and macrophage colony-stimulating factor receptors play distinct roles in late-stage myeloid cell differentiation. Blood.

[64] Hohaus S., Petrovick M.S., Voso M.T., Sun Z., Zhang D.E., Tenen D.G. (1995). PU.1 (Spi-1) and C/EBP alpha regulate expression of the granulocyte-macrophage colony-stimulating factor receptor alpha gene. Mol. Cell. Biol..

[65] Smith L.T., Hohaus S., Gonzalez D.A., Dziennis S.E., Tenen D.G. (1996). PU.1 (Spi-1) and C/EBP alpha regulate the granulocyte colony-stimulating factor receptor promoter in myeloid cells. Blood.

[66] Okuno Y., Huang G., Rosenbauer F., Evans E.K., Radomska H.S., Iwasaki H., Akashi K., Moreau-Gachelin F., Li Y., Zhang P. (2005). Potential autoregulation of transcription factor PU.1 by an upstream regulatory element. Mol. Cell. Biol..

[67] Schuetzmann D., Walter C., van Riel B., Kruse S., Konig T., Erdmann T., Tonges A., Bindels E., Weilemann A., Gebhard C. (2018). Temporal autoregulation during human PU.1 locus SubTAD formation. Blood.

[68] Kueh H.Y., Champhekar A., Nutt S.L., Elowitz M.B., Rothenberg E.V. (2013). Positive feedback between PU.1 and the cell cycle controls myeloid differentiation. Science.

[69] Rosa A., Ballarino M., Sorrentino A., Sthandier O., De Angelis F.G., Marchioni M., Masella B., Guarini A., Fatica A., Peschle C. (2007). The interplay between the master transcription factor PU.1 and miR-424 regulates human monocyte/macrophage differentiation. Proc. Natl. Acad. Sci. USA.

[70] Trinh B.Q., Ummarino S., Zhang Y., Ebralidze A.K., Bassal M.A., Nguyen T.M., Heller G., Coffey R., Tenen D.E., van der Kouwe E. (2021). Myeloid lncRNA *LOUP* mediates opposing regulatory effects of RUNX1 and RUNX1-ETO in t(8;21) AML. Blood.

[71] Halasz H., Malekos E., Covarrubias S., Yitiz S., Montano C., Sudek L., Katzman S., Liu S.J., Horlbeck M.A., Namvar L. (2024). CRISPRi screens identify the lncRNA, *LOUP*, as a multifunctional locus regulating macrophage differentiation and inflammatory signaling. Proc. Natl. Acad. Sci. USA.

[72] Joo M., Kwon M., Cho Y.J., Hu N., Pedchenko T.V., Sadikot R.T., Blackwell T.S., Christman J.W. (2009). Lipopolysaccharide-dependent interaction between PU.1 and c-Jun determines production of lipocalin-type prostaglandin D synthase and prostaglandin D2 in macrophages. Am. J. Physiol. Lung Cell. Mol. Physiol..

[73] Kim S., Bagadia P., Anderson D.A., Liu T.T., Huang X., Theisen D.J., O’Connor K.W., Ohara R.A., Iwata A., Murphy T.L. (2020). High Amount of Transcription Factor IRF8 Engages AP1-IRF Composite Elements in Enhancers to Direct Type 1 Conventional Dendritic Cell Identity. Immunity.

[74] Schonheit J., Kuhl C., Gebhardt M.L., Klett F.F., Riemke P., Scheller M., Huang G., Naumann R., Leutz A., Stocking C. (2013). PU.1 level-directed chromatin structure remodeling at the *Irf8* gene drives dendritic cell commitment. Cell Rep..

[75] Lian T., Guan R., Zhou B.R., Bai Y. (2024). Structural mechanism of synergistic targeting of the CX3CR1 nucleosome by PU.1 and C/EBPalpha. Nat. Struct. Mol. Biol..

[76] Chambers C., Cermakova K., Chan Y.S., Kurtz K., Wohlan K., Lewis A.H., Wang C., Pham A., Dejmek M., Sala M. (2023). SWI/SNF Blockade Disrupts PU.1-Directed Enhancer Programs in Normal Hematopoietic Cells and Acute Myeloid Leukemia. Cancer Res..

[77] Tagore M., McAndrew M.J., Gjidoda A., Floer M. (2015). The Lineage-Specific Transcription Factor PU.1 Prevents Polycomb-Mediated Heterochromatin Formation at Macrophage-Specific Genes. Mol. Cell. Biol..

[78] Choubey P., Kumar A., Podder S., Raghav S.K., Bansal K. (2026). Cohesin complex cooperates with PU.1 at super-enhancers to regulate the differentiation and identity of conventional dendritic cells. Cell Rep..

[79] Watt S., Vasquez L., Walter K., Mann A.L., Kundu K., Chen L., Sims Y., Ecker S., Burden F., Farrow S. (2021). Genetic perturbation of PU.1 binding and chromatin looping at neutrophil enhancers associates with autoimmune disease. Nat. Commun..

[80] DeKoter R.P., Walsh J.C., Singh H. (1998). PU.1 regulates both cytokine-dependent proliferation and differentiation of granulocyte/macrophage progenitors. EMBO J..

[81] Iwasaki H., Somoza C., Shigematsu H., Duprez E.A., Iwasaki-Arai J., Mizuno S., Arinobu Y., Geary K., Zhang P., Dayaram T. (2005). Distinctive and indispensable roles of PU.1 in maintenance of hematopoietic stem cells and their differentiation. Blood.

[82] Karpurapu M., Wang X., Deng J., Park H., Xiao L., Sadikot R.T., Frey R.S., Maus U.A., Park G.Y., Scott E.W. (2011). Functional PU.1 in macrophages has a pivotal role in NF-kappaB activation and neutrophilic lung inflammation during endotoxemia. Blood.

[83] Fisher R.C., Olson M.C., Pongubala J.M., Perkel J.M., Atchison M.L., Scott E.W., Simon M.C. (1998). Normal myeloid development requires both the glutamine-rich transactivation domain and the PEST region of transcription factor PU.1 but not the potent acidic transactivation domain. Mol. Cell. Biol..

[84] Rangatia J., Vangala R.K., Treiber N., Zhang P., Radomska H., Tenen D.G., Hiddemann W., Behre G. (2002). Downregulation of *c-Jun* expression by transcription factor C/EBPalpha is critical for granulocytic lineage commitment. Mol. Cell. Biol..

[85] Wang H., Jain S., Li P., Lin J.X., Oh J., Qi C., Gao Y., Sun J., Sakai T., Naghashfar Z. (2019). Transcription factors IRF8 and PU.1 are required for follicular B cell development and BCL6-driven germinal center responses. Proc. Natl. Acad. Sci. USA.

[86] Smith M.A., Wright G., Wu J., Tailor P., Ozato K., Chen X., Wei S., Piskurich J.F., Ting J.P., Wright K.L. (2011). Positive regulatory domain I (PRDM1) and IRF8/PU.1 counter-regulate MHC class II transactivator (CIITA) expression during dendritic cell maturation. J. Biol. Chem..

[87] Meraro D., Gleit-Kielmanowicz M., Hauser H., Levi B.Z. (2002). IFN-stimulated gene 15 is synergistically activated through interactions between the myelocyte/lymphocyte-specific transcription factors, PU.1, IFN regulatory factor-8/IFN consensus sequence binding protein, and IFN regulatory factor-4: Characterization of a new subtype of IFN-stimulated response element. J. Immunol..

[88] Cha M.S., Kang M.H., Lee J., Hong J., Jeong Y.S., Bae Y.S., Lee H., Park S.H., Bae Y.S. (2026). TopBP1 orchestrates PU.1-IRF8 transcriptional programming of dendritic cell differentiation and Flt3L-driven tumor immunity. Exp. Mol. Med..

[89] Eisenbeis C.F., Singh H., Storb U. (1995). Pip, a novel IRF family member, is a lymphoid-specific, PU.1-dependent transcriptional activator. Genes Dev..

[90] Carotta S., Willis S.N., Hasbold J., Inouye M., Pang S.H., Emslie D., Light A., Chopin M., Shi W., Wang H. (2014). The transcription factors IRF8 and PU.1 negatively regulate plasma cell differentiation. J. Exp. Med..

[91] Willcockson M.A., Taylor S.J., Ghosh S., Healton S.E., Wheat J.C., Wilson T.J., Steidl U., Skoultchi A.I. (2019). Runx1 promotes murine erythroid progenitor proliferation and inhibits differentiation by preventing Pu.1 downregulation. Proc. Natl. Acad. Sci. USA.

[92] Huang G., Zhang P., Hirai H., Elf S., Yan X., Chen Z., Koschmieder S., Okuno Y., Dayaram T., Growney J.D. (2008). PU.1 is a major downstream target of AML1 (RUNX1) in adult mouse hematopoiesis. Nat. Genet..

[93] Gu X., Hu Z., Ebrahem Q., Crabb J.S., Mahfouz R.Z., Radivoyevitch T., Crabb J.W., Saunthararajah Y. (2014). Runx1 regulation of Pu.1 corepressor/coactivator exchange identifies specific molecular targets for leukemia differentiation therapy. J. Biol. Chem..

[94] Rodriguez-Perales S., Torres-Ruiz R., Suela J., Acquadro F., Martin M.C., Yebra E., Ramirez J.C., Alvarez S., Cigudosa J.C. (2016). Truncated RUNX1 protein generated by a novel t(1;21)(p32;q22) chromosomal translocation impairs the proliferation and differentiation of human hematopoietic progenitors. Oncogene.

[95] Bashir H.S., Binod G.C., Rahman A.A., Dong F. (2025). A critical role of the Erk1/2 downstream targets *c-Fos*, *Egr-1*, and *Egr-2* in PU.1-driven monocyte development. J. Leukoc. Biol..

[96] Nerlov C., Querfurth E., Kulessa H., Graf T. (2000). GATA-1 interacts with the myeloid PU.1 transcription factor and represses PU.1-dependent transcription. Blood.

[97] Zhang P., Zhang X., Iwama A., Yu C., Smith K.A., Mueller B.U., Narravula S., Torbett B.E., Orkin S.H., Tenen D.G. (2000). PU.1 inhibits GATA-1 function and erythroid differentiation by blocking GATA-1 DNA binding. Blood.

[98] Burda P., Vargova J., Curik N., Salek C., Papadopoulos G.L., Strouboulis J., Stopka T. (2016). GATA-1 Inhibits *PU.1* Gene via DNA and Histone H3K9 Methylation of Its Distal Enhancer in Erythroleukemia. PLoS ONE.

[99] Chou S.T., Khandros E., Bailey L.C., Nichols K.E., Vakoc C.R., Yao Y., Huang Z., Crispino J.D., Hardison R.C., Blobel G.A. (2009). Graded repression of *PU.1/Sfpi1* gene transcription by GATA factors regulates hematopoietic cell fate. Blood.

[100] Nerlov C., Graf T. (1998). PU.1 induces myeloid lineage commitment in multipotent hematopoietic progenitors. Genes Dev..

[101] Xie H., Ye M., Feng R., Graf T. (2004). Stepwise reprogramming of B cells into macrophages. Cell.

[102] Laiosa C.V., Stadtfeld M., Xie H., de Andres-Aguayo L., Graf T. (2006). Reprogramming of committed T cell progenitors to macrophages and dendritic cells by C/EBP alpha and PU.1 transcription factors. Immunity.

[103] Etzrodt M., Ahmed N., Hoppe P.S., Loeffler D., Skylaki S., Hilsenbeck O., Kokkaliaris K.D., Kaltenbach H.M., Stelling J., Nerlov C. (2019). Inflammatory signals directly instruct PU.1 in HSCs via TNF. Blood.

[104] Le Coz C., Nguyen D.N., Su C., Nolan B.E., Albrecht A.V., Xhani S., Sun D., Demaree B., Pillarisetti P., Khanna C. (2021). Constrained chromatin accessibility in PU.1-mutated agammaglobulinemia patients. J. Exp. Med..

[105] Arinobu Y., Mizuno S., Chong Y., Shigematsu H., Iino T., Iwasaki H., Graf T., Mayfield R., Chan S., Kastner P. (2007). Reciprocal activation of GATA-1 and PU.1 marks initial specification of hematopoietic stem cells into myeloerythroid and myelolymphoid lineages. Cell Stem Cell.

[106] Burda P., Laslo P., Stopka T. (2010). The role of PU.1 and GATA-1 transcription factors during normal and leukemogenic hematopoiesis. Leukemia.

[107] Anderson M.K., Weiss A.H., Hernandez-Hoyos G., Dionne C.J., Rothenberg E.V. (2002). Constitutive expression of PU.1 in fetal hematopoietic progenitors blocks T cell development at the pro-T cell stage. Immunity.

[108] Dakic A., Metcalf D., Di Rago L., Mifsud S., Wu L., Nutt S.L. (2005). PU.1 regulates the commitment of adult hematopoietic progenitors and restricts granulopoiesis. J. Exp. Med..

[109] Pang S.H.M., de Graaf C.A., Hilton D.J., Huntington N.D., Carotta S., Wu L., Nutt S.L. (2018). PU.1 Is Required for the Developmental Progression of Multipotent Progenitors to Common Lymphoid Progenitors. Front. Immunol..

[110] Zong L., Park B., Tekin-Turhan F., Kuribayashi W., Tanaka-Yano M., Li K., Beerman I. (2026). Epigenetic profiling of hematopoietic stem cells from male mice identifies KDR and PU.1 as regulators of aging transcriptome and caloric restriction response. Nat. Commun..

[111] Dahl R., Walsh J.C., Lancki D., Laslo P., Iyer S.R., Singh H., Simon M.C. (2003). Regulation of macrophage and neutrophil cell fates by the PU.1:C/EBPalpha ratio and granulocyte colony-stimulating factor. Nat. Immunol..

[112] Feng R., Desbordes S.C., Xie H., Tillo E.S., Pixley F., Stanley E.R., Graf T. (2008). PU.1 and C/EBPalpha/beta convert fibroblasts into macrophage-like cells. Proc. Natl. Acad. Sci. USA.

[113] Sevilla L., Zaldumbide A., Carlotti F., Dayem M.A., Pognonec P., Boulukos K.E. (2001). *Bcl-XL* expression correlates with primary macrophage differentiation, activation of functional competence, and survival and results from synergistic transcriptional activation by Ets2 and PU.1. J. Biol. Chem..

[114] Berclaz P.Y., Zsengeller Z., Shibata Y., Otake K., Strasbaugh S., Whitsett J.A., Trapnell B.C. (2002). Endocytic internalization of adenovirus, nonspecific phagocytosis, and cytoskeletal organization are coordinately regulated in alveolar macrophages by GM-CSF and PU.1. J. Immunol..

[115] Lin Q., Chauvistre H., Costa I.G., Gusmao E.G., Mitzka S., Hanzelmann S., Baying B., Klisch T., Moriggl R., Hennuy B. (2015). Epigenetic program and transcription factor circuitry of dendritic cell development. Nucleic Acids Res..

[116] Kitamura N., Yokoyama H., Yashiro T., Nakano N., Nishiyama M., Kanada S., Fukai T., Hara M., Ikeda S., Ogawa H. (2012). Role of PU.1 in MHC class II expression through transcriptional regulation of class II transactivator pI in dendritic cells. J. Allergy Clin. Immunol..

[117] Kanada S., Nishiyama C., Nakano N., Suzuki R., Maeda K., Hara M., Kitamura N., Ogawa H., Okumura K. (2011). Critical role of transcription factor PU.1 in the expression of CD80 and CD86 on dendritic cells. Blood.

[118] Chopin M., Lun A.T., Zhan Y., Schreuder J., Coughlan H., D’Amico A., Mielke L.A., Almeida F.F., Kueh A.J., Dickins R.A. (2019). Transcription Factor PU.1 Promotes Conventional Dendritic Cell Identity and Function via Induction of Transcriptional Regulator DC-SCRIPT. Immunity.

[119] Fischer J., Walter C., Tonges A., Aleth H., Jordao M.J.C., Leddin M., Groning V., Erdmann T., Lenz G., Roth J. (2019). Safeguard function of PU.1 shapes the inflammatory epigenome of neutrophils. Nat. Immunol..

[120] Anderson K.L., Smith K.A., Pio F., Torbett B.E., Maki R.A. (1998). Neutrophils deficient in PU.1 do not terminally differentiate or become functionally competent. Blood.

[121] Oelgeschlager M., Nuchprayoon I., Luscher B., Friedman A.D. (1996). C/EBP, c-Myb, and PU.1 cooperate to regulate the neutrophil elastase promoter. Mol. Cell. Biol..

[122] Ford A.M., Bennett C.A., Healy L.E., Towatari M., Greaves M.F., Enver T. (1996). Regulation of the myeloperoxidase enhancer binding proteins Pu1, C-EBP alpha, -beta, and -delta during granulocyte-lineage specification. Proc. Natl. Acad. Sci. USA.

[123] Polli M., Dakic A., Light A., Wu L., Tarlinton D.M., Nutt S.L. (2005). The development of functional B lymphocytes in conditional PU.1 knock-out mice. Blood.

[124] Hosokawa H., Koizumi M., Masuhara K., Romero-Wolf M., Tanaka T., Nakayama T., Rothenberg E.V. (2021). Stage-specific action of Runx1 and GATA3 controls silencing of *PU.1* expression in mouse pro-T cells. J. Exp. Med..

[125] Champhekar A., Damle S.S., Freedman G., Carotta S., Nutt S.L., Rothenberg E.V. (2015). Regulation of early T-lineage gene expression and developmental progression by the progenitor cell transcription factor PU.1. Genes Dev..

[126] Wei Z., Pan X., Cui X., Zhang J., Dai X., Zeng Y., Chen X. (2025). PU.1 dictates beta-amyloid-induced *TREM2* expression upregulation in microglia in a transgenic model of Alzheimer’s disease. Front. Aging Neurosci..

[127] Cakir B., Tanaka Y., Kiral F.R., Xiang Y., Dagliyan O., Wang J., Lee M., Greaney A.M., Yang W.S., duBoulay C. (2022). Expression of the transcription factor PU.1 induces the generation of microglia-like cells in human cortical organoids. Nat. Commun..

[128] Smith A.M., Gibbons H.M., Oldfield R.L., Bergin P.M., Mee E.W., Faull R.L., Dragunow M. (2013). The transcription factor PU.1 is critical for viability and function of human brain microglia. Glia.

[129] Rustenhoven J., Smith A.M., Smyth L.C., Jansson D., Scotter E.L., Swanson M.E.V., Aalderink M., Coppieters N., Narayan P., Handley R. (2018). PU.1 regulates Alzheimer’s disease-associated genes in primary human microglia. Mol. Neurodegener..

[130] Knox A.V.C., Cominsky L.Y., Sun D., Cruz Cabrera E., Nolan B.E., Ofray E., Benetti E., Visconti C., Barzaghi F., Rosenzweig S.D. (2025). One hundred thirty-four germ line *PU.1* variants and the agammaglobulinemic patients carrying them. Blood.

[131] Cook W.D., McCaw B.J., Herring C., John D.L., Foote S.J., Nutt S.L., Adams J.M. (2004). PU.1 is a suppressor of myeloid leukemia, inactivated in mice by gene deletion and mutation of its DNA binding domain. Blood.

[132] Zhou J., Wu J., Li B., Liu D., Yu J., Yan X., Zheng S., Wang J., Zhang L., Zhang L. (2014). PU.1 is essential for MLL leukemia partially via crosstalk with the MEIS/HOX pathway. Leukemia.

[133] Aikawa Y., Yamagata K., Katsumoto T., Shima Y., Shino M., Stanley E.R., Cleary M.L., Akashi K., Tenen D.G., Kitabayashi I. (2015). Essential role of PU.1 in maintenance of mixed lineage leukemia-associated leukemic stem cells. Cancer Sci..

[134] Sive J.I., Basilico S., Hannah R., Kinston S.J., Calero-Nieto F.J., Gottgens B. (2016). Genome-scale definition of the transcriptional programme associated with compromised PU.1 activity in acute myeloid leukaemia. Leukemia.

[135] Durual S., Rideau A., Ruault-Jungblut S., Cossali D., Beris P., Piguet V., Matthes T. (2007). Lentiviral PU.1 overexpression restores differentiation in myeloid leukemic blasts. Leukemia.

[136] Mueller B.U., Pabst T., Osato M., Asou N., Johansen L.M., Minden M.D., Behre G., Hiddemann W., Ito Y., Tenen D.G. (2002). Heterozygous *PU.1* mutations are associated with acute myeloid leukemia. Blood.

[137] Suraweera N., Meijne E., Moody J., Carvajal-Carmona L.G., Yoshida K., Pollard P., Fitzgibbon J., Riches A., van Laar T., Huiskamp R. (2005). Mutations of the *PU.1* Ets domain are specifically associated with murine radiation-induced, but not human therapy-related, acute myeloid leukaemia. Oncogene.

[138] Ley T.J., Miller C., Ding L., Raphael B.J., Mungall A.J., Robertson A., Hoadley K., Triche T.J., Laird P.W., The Cancer Genome Atlas Research Network (2013). Genomic and epigenomic landscapes of adult de novo acute myeloid leukemia. N. Engl. J. Med..

[139] Papaemmanuil E., Gerstung M., Bullinger L., Gaidzik V.I., Paschka P., Roberts N.D., Potter N.E., Heuser M., Thol F., Bolli N. (2016). Genomic Classification and Prognosis in Acute Myeloid Leukemia. N. Engl. J. Med..

[140] Vangala R.K., Heiss-Neumann M.S., Rangatia J.S., Singh S.M., Schoch C., Tenen D.G., Hiddemann W., Behre G. (2003). The myeloid master regulator transcription factor PU.1 is inactivated by AML1-ETO in t(8;21) myeloid leukemia. Blood.

[141] Wang K., Wang P., Shi J., Zhu X., He M., Jia X., Yang X., Qiu F., Jin W., Qian M. (2010). PML/RARalpha targets promoter regions containing *PU.1* consensus and RARE half sites in acute promyelocytic leukemia. Cancer Cell.

[142] Hong J., Sui P., Li Y., Xu K.Y., Lee J.H., Wang J., Chen S., Zhang P., Wingate N., Noor A. (2025). PSPC1 exerts an oncogenic role in AML by regulating a leukemic transcription program in cooperation with PU.1. Cell Stem Cell.

[143] Staber P.B., Zhang P., Ye M., Welner R.S., Levantini E., Di Ruscio A., Ebralidze A.K., Bach C., Zhang H., Zhang J. (2014). The Runx-PU.1 pathway preserves normal and AML/ETO9a leukemic stem cells. Blood.

[144] Steidl U., Steidl C., Ebralidze A., Chapuy B., Han H.J., Will B., Rosenbauer F., Becker A., Wagner K., Koschmieder S. (2007). A distal single nucleotide polymorphism alters long-range regulation of the *PU.1* gene in acute myeloid leukemia. J. Clin. Investig..

[145] Bonadies N., Pabst T., Mueller B.U. (2010). Heterozygous deletion of the *PU.1* locus in human AML. Blood.

[146] Rosenbauer F., Owens B.M., Yu L., Tumang J.R., Steidl U., Kutok J.L., Clayton L.K., Wagner K., Scheller M., Iwasaki H. (2006). Lymphoid cell growth and transformation are suppressed by a key regulatory element of the gene encoding PU.1. Nat. Genet..

[147] Gerloff D., Grundler R., Wurm A.A., Brauer-Hartmann D., Katzerke C., Hartmann J.U., Madan V., Muller-Tidow C., Duyster J., Tenen D.G. (2015). NF-kappaB/STAT5/miR-155 network targets *PU.1* in FLT3-ITD-driven acute myeloid leukemia. Leukemia.

[148] Basova P., Pospisil V., Savvulidi F., Burda P., Vargova K., Stanek L., Dluhosova M., Kuzmova E., Jonasova A., Steidl U. (2014). Aggressive acute myeloid leukemia in *PU.1/p53* double-mutant mice. Oncogene.

[149] Yeamans C., Wang D., Paz-Priel I., Torbett B.E., Tenen D.G., Friedman A.D. (2007). C/EBPalpha binds and activates the *PU.1* distal enhancer to induce monocyte lineage commitment. Blood.

[150] Huang G., Zhao X., Wang L., Elf S., Xu H., Zhao X., Sashida G., Zhang Y., Liu Y., Lee J. (2011). The ability of MLL to bind RUNX1 and methylate H3K4 at *PU.1* regulatory regions is impaired by MDS/AML-associated *RUNX1/AML1* mutations. Blood.

[151] Curik N., Burda P., Vargova K., Pospisil V., Belickova M., Vlckova P., Savvulidi F., Necas E., Hajkova H., Haskovec C. (2012). 5-azacitidine in aggressive myelodysplastic syndromes regulates chromatin structure at *PU.1* gene and cell differentiation capacity. Leukemia.

[152] Cheng J.X., Anastasi J., Watanabe K., Kleinbrink E.L., Grimley E., Knibbs R., Shen Q.J., Vardiman J.W. (2013). Genome-wide profiling reveals epigenetic inactivation of the PU.1 pathway by histone H3 lysine 27 trimethylation in cytogenetically normal myelodysplastic syndrome. Leukemia.

[153] Endo S., Nishimura N., Toyoda K., Komohara Y., Carreras J., Yuki H., Shichijo T., Ueno S., Ueno N., Hirata S. (2024). Decreased *PU.1* expression in mature B cells induces lymphomagenesis. Cancer Sci..

[154] Sokalski K.M., Li S.K., Welch I., Cadieux-Pitre H.A., Gruca M.R., DeKoter R.P. (2011). Deletion of genes encoding PU.1 and Spi-B in B cells impairs differentiation and induces pre-B cell acute lymphoblastic leukemia. Blood.

[155] Tatetsu H., Ueno S., Hata H., Yamada Y., Takeya M., Mitsuya H., Tenen D.G., Okuno Y. (2007). Down-regulation of PU.1 by methylation of distal regulatory elements and the promoter is required for myeloma cell growth. Cancer Res..

[156] Huskova H., Korecka K., Karban J., Vargova J., Vargova K., Dusilkova N., Trneny M., Stopka T. (2015). Oncogenic microRNA-155 and its target PU.1: An integrative gene expression study in six of the most prevalent lymphomas. Int. J. Hematol..

[157] Yuki H., Ueno S., Tatetsu H., Niiro H., Iino T., Endo S., Kawano Y., Komohara Y., Takeya M., Hata H. (2013). PU.1 is a potent tumor suppressor in classical Hodgkin lymphoma cells. Blood.

[158] Dluhosova M., Curik N., Vargova J., Jonasova A., Zikmund T., Stopka T. (2014). Epigenetic control of *SPI1* gene by CTCF and ISWI ATPase SMARCA5. PLoS ONE.

[159] Liu Z., Qin Z., Li H., Zhu L., He L., Chen N., Zhu D., Liu Q., Dai L., Peng X. (2026). *Spi-1* proto-oncogene regulates mRNA hypertranscription and malignant progression in head and neck cancer. Signal Transduct. Target. Ther..

[160] Huang J., Chen W., Jie Z., Jiang M. (2022). Comprehensive Analysis of Immune Implications and Prognostic Value of *SPI1* in Gastric Cancer. Front. Oncol..

[161] Liu C., Qiu X., Gao J., Gong Z., Zhou X., Luo H., Geng X. (2023). *SPI1* involvement in malignant melanoma pathogenesis by regulation of HK2 through the AKT1/mTOR pathway. J. Cell. Mol. Med..

[162] Chen F., Yu L., Wang D., Zhao Y., Li C. (2026). *SPI1* mediates CST2 transcription to promote the proliferation, metastasis, and angiogenesis of esophageal cancer. Arch. Biochem. Biophys..

[163] Yang J., Liu C., Guan J., Wang Y., Su J., Wang Y., Liu S. (2022). *SPI1* mediates transcriptional activation of TPX2 and RNF2 to regulate the radiosensitivity of lung squamous cell carcinoma. Arch. Biochem. Biophys..

[164] Lin J., Liu W., Luan T., Yuan L., Jiang W., Cai H., Yuan W., Wang Y., Zhang Q., Wang L. (2017). High expression of *PU.1* is associated with Her-2 and shorter survival in patients with breast cancer. Oncol. Lett..

[165] Bonollo F., Hanhart D., de Brot S., Cheng W., Chouvardas P., Roth B., Thalmann G.N., Sampson N., Kruithof-de Julio M., Karkampouna S. (2026). PU.1 is a mediator of the reactive stromal response associated with castration-resistant prostate cancer. Commun. Biol..

[166] Eskandarian Boroujeni M., Lopacinska N., Antonczyk A., Kluzek K., Wesoly J., Bluyssen H.A.R. (2025). Integrative multi-omics analysis of IFNgamma-induced macrophages and atherosclerotic plaques reveals macrophage-dependent STAT1-driven transcription in atherosclerosis. Front. Immunol..

[167] Park S.Y., Lee S.W., Kim H.Y., Lee S.Y., Lee W.S., Hong K.W., Kim C.D. (2016). SIRT1 inhibits differentiation of monocytes to macrophages: Amelioration of synovial inflammation in rheumatoid arthritis. J. Mol. Med..

[168] Liu C., Wang M., Sun W., Cai F., Geng S., Su X., Shi Y. (2017). PU.1 serves a critical role in the innate defense against Aspergillus fumigatus via dendritic cell-associated C-type lectin receptor-1 and toll-like receptors-2 and 4 in THP-1-derived macrophages. Mol. Med. Rep..

[169] Carter M.J., Bogdanov Y.D., Smith R.C., Cox K.L., Frampton S., Ferson L., Foxall R.B., Hussain K., Strefford J.C., Beers S.A. (2025). The ETS-family transcription factor PU.1 is a critical regulator of the inhibitory Fcgamma receptor IIB expression in humans. J. Immunol..

[170] Horvai A., Palinski W., Wu H., Moulton K.S., Kalla K., Glass C.K. (1995). Scavenger receptor A gene regulatory elements target gene expression to macrophages and to foam cells of atherosclerotic lesions. Proc. Natl. Acad. Sci. USA.

[171] Inaba T., Gotoda T., Ishibashi S., Harada K., Ohsuga J.I., Ohashi K., Yazaki Y., Yamada N. (1996). Transcription factor PU.1 mediates induction of *c-fms* in vascular smooth muscle cells: A mechanism for phenotypic change to phagocytic cells. Mol. Cell. Biol..

[172] Azizan A., Farhadi E., Faezi S.T., Alikhani M., Jamshidi A., Vodjgani M., Mahmoudi M. (2025). Overexpression of *IRF8* and *Pu.1* in B cells of systemic lupus erythematosus patients. Lupus.

[173] Shakerian L., Ghorbani S., Talebi F., Noorbakhsh F. (2018). MicroRNA-150 targets PU.1 and regulates macrophage differentiation and function in experimental autoimmune encephalomyelitis. J. Neuroimmunol..

[174] Jin L., Pei S., Dong L., Li X., Kuang Y., Zhu W., Chen X., Yin M. (2026). *PU.1* drives psoriasis pathogenesis by promoting intermediate monocytes-to-M1 macrophage differentiation through Dectin-1 signaling. J. Autoimmun..

[175] Fu M., Liu Y., Jing Y., Wang J. (2026). Synovial resident macrophage-derived PU.1 alleviates rheumatoid arthritis by promoting efferocytosis of fibroblast-like synoviocytes. Clin. Exp. Rheumatol..

[176] Chavez J.S., Rabe J.L., Nino K.E., Wells H.H., Gessner R.L., Mills T.S., Hernandez G., Pietras E.M. (2023). PU.1 is required to restrain myelopoiesis during chronic inflammatory stress. Front. Cell Dev. Biol..

[177] Wohlfahrt T., Rauber S., Uebe S., Luber M., Soare A., Ekici A., Weber S., Matei A.E., Chen C.W., Maier C. (2019). PU.1 controls fibroblast polarization and tissue fibrosis. Nature.

[178] Liu Q., Yu J., Wang L., Tang Y., Zhou Q., Ji S., Wang Y., Santos L., Haeusler R.A., Que J. (2020). Inhibition of PU.1 ameliorates metabolic dysfunction and non-alcoholic steatohepatitis. J. Hepatol..

[179] Hu J., Zhang J.J., Li L., Wang S.L., Yang H.T., Fan X.W., Zhang L.M., Hu G.L., Fu H.X., Song W.F. (2021). PU.1 inhibition attenuates atrial fibrosis and atrial fibrillation vulnerability induced by angiotensin-II by reducing TGF-beta1/Smads pathway activation. J. Cell. Mol. Med..

[180] Kim B., Tate M.D., Karahan H., Wijeratne H.R.S., Sharify A.D., Wang S.S., Kim J.R., Al-Amin M.M., Hartigan K., Chung S. (2026). Deletion of *SPI1* in microglia exacerbates amyloid pathology by impairing microglial response in Alzheimer’s disease models. Neuron.

[181] Wu Y., Guo W., Kuang H., Wu X., Trinh T.H., Wang Y., Zhao S., Wen Z., Yu T. (2025). *Pu.1/Spi1* dosage controls the turnover and maintenance of microglia in zebrafish and mammals. eLife.

[182] Ayata P., Crowley J.M., Challman M.F., Sahasrabuddhe V., Gratuze M., Werneburg S., Ribeiro D., Hays E.C., Duran-Laforet V., Faust T.E. (2025). Lymphoid gene expression supports neuroprotective microglia function. Nature.

[183] Zhou N., Liu K., Sun Y., Cao Y., Yang J. (2019). Transcriptional mechanism of *IRF8* and *PU.1* governs microglial activation in neurodegenerative condition. Protein Cell.

[184] Pradhan R., Sakib M.S., Kaurani L., Kruger D.M., Pena T., Burkhardt S., Schutz A.L., Kronenberg-Versteeg D., Delalle I., Sananbenesi F. (2026). LncRNA *Glelr* modulates microglia inflammatory programs in association with PU.1. Neurobiol. Dis..

[185] Ljubikj T., Mars M.Z., van der Geest A.T., Jakobs C.E., Bessler N., Donega V., van den Oetelaar X., de Wit M., Pasterkamp R.J. (2026). PU.1 restores microglial dysfunction caused by *C9ORF72* repeat expansions in neural organoids. Brain.

[186] Masrori P., Bijnens B., Fumagalli L., Davie K., Poovathingal S.K., Meese T., Storm A., Hersmus N., Fazal R., van den Biggelaar D. (2025). *C9orf72* hexanucleotide repeat expansions impair microglial response in ALS. Nat. Neurosci..

[187] Ralvenius W.T., Andresen J.L., Huston M.M., Penney J., Bonner J.M., Fenton O.S., Langer R., Tsai L.H. (2024). Nanoparticle-Mediated Delivery of Anti-*PU.1* siRNA via Localized Intracisternal Administration Reduces Neuroinflammation. Adv. Mater..

[188] Antony-Debre I., Paul A., Leite J., Mitchell K., Kim H.M., Carvajal L.A., Todorova T.I., Huang K., Kumar A., Farahat A.A. (2017). Pharmacological inhibition of the transcription factor PU.1 in leukemia. J. Clin. Investig..

[189] Taylor S.J., Stauber J., Bohorquez O., Tatsumi G., Kumari R., Chakraborty J., Bartholdy B.A., Schwenger E., Sundaravel S., Farahat A.A. (2024). Pharmacological restriction of genomic binding sites redirects PU.1 pioneer transcription factor activity. Nat. Genet..

[190] Roe J.S., Mercan F., Rivera K., Pappin D.J., Vakoc C.R. (2015). BET Bromodomain Inhibition Suppresses the Function of Hematopoietic Transcription Factors in Acute Myeloid Leukemia. Mol. Cell.

[191] Cusan M., Cai S.F., Mohammad H.P., Krivtsov A., Chramiec A., Loizou E., Witkin M.D., Smitheman K.N., Tenen D.G., Ye M. (2018). LSD1 inhibition exerts its antileukemic effect by recommissioning PU.1- and C/EBPalpha-dependent enhancers in AML. Blood.

[192] Lau S.F., Chen C., Fu W.Y., Qu J.Y., Cheung T.H., Fu A.K.Y., Ip N.Y. (2020). IL-33-*PU.1* Transcriptome Reprogramming Drives Functional State Transition and Clearance Activity of Microglia in Alzheimer’s Disease. Cell Rep..

[193] Shi D., Toyonaga S., Anderson D.G. (2023). In Vivo RNA Delivery to Hematopoietic Stem and Progenitor Cells via Targeted Lipid Nanoparticles. Nano Lett..

[194] Ramishetti S., Hazan-Halevy I., Palakuri R., Chatterjee S., Naidu Gonna S., Dammes N., Freilich I., Kolik Shmuel L., Danino D., Peer D. (2020). A Combinatorial Library of Lipid Nanoparticles for RNA Delivery to Leukocytes. Adv. Mater..

[195] Cao M., Fay F., Hotier A., Domenichini S., Alexandre L., Ribes C., Gazeau F., Hillaireau H., Fattal E. (2026). Selective mRNA Delivery to Activated Macrophages via Hyaluronic Acid-Functionalized Lipid Nanoparticles with Optimized PEGylation. Biomacromolecules.

[196] Papp T.E., Zeng J., Shahnawaz H., Akyianu A., Breda L., Yadegari A., Steward J., Shi R., Li Q., Mui B.L. (2025). CD47 peptide-cloaked lipid nanoparticles promote cell-specific mRNA delivery. Mol. Ther..

[197] Casimiro L.D.M., Escudero R., du Plessis M., Schiffelers R., Heidenreich O. (2026). Lipid nanoparticles for bone marrow-targeted RNA therapeutics. J. Nanobiotechnol..

[198] Zhong G., Long H., Zhou T., Liu Y., Zhao J., Han J., Yang X., Yu Y., Chen F., Shi S. (2022). Blood-brain barrier Permeable nanoparticles for Alzheimer’s disease treatment by selective mitophagy of microglia. Biomaterials.

[199] Wang P., Yang P., Qian K., Li Y., Xu S., Meng R., Guo Q., Cheng Y., Cao J., Xu M. (2022). Precise gene delivery systems with detachable albumin shell remodeling dysfunctional microglia by *TREM2* for treatment of Alzheimer’s disease. Biomaterials.

[200] Zhou L., Wang Y., Xu Y., Zhang Y., Zhu C. (2024). A comprehensive review of AAV-mediated strategies targeting microglia for therapeutic intervention of neurodegenerative diseases. J. Neuroinflammation.

[201] Zhang Q., Liu Y., Zhang J., Li Y., Wang J., Liu N., Zhang J., Pan X. (2024). Discovery of novel penetrating peptides able to target human leukemia and lymphoma for enhanced PROTAC delivery. Eur. J. Med. Chem..

[202] He Y., Khan S., Huo Z., Lv D., Zhang X., Liu X., Yuan Y., Hromas R., Xu M., Zheng G. (2020). Proteolysis targeting chimeras (PROTACs) are emerging therapeutics for hematologic malignancies. J. Hematol. Oncol..

